# Healthy lifestyle behaviors, mediating biomarkers, and risk of microvascular complications among individuals with type 2 diabetes: A cohort study

**DOI:** 10.1371/journal.pmed.1004135

**Published:** 2023-01-10

**Authors:** Tingting Geng, Kai Zhu, Qi Lu, Zhenzhen Wan, Xue Chen, Liegang Liu, An Pan, Gang Liu

**Affiliations:** 1 Department of Nutrition and Food Hygiene, Hubei Key Laboratory of Food Nutrition and Safety, Ministry of Education Key Lab of Environment and Health, and State Key Laboratory of Environment Health (Incubating), School of Public Health, Tongji Medical College, Huazhong University of Science and Technology, Wuhan, China; 2 Department of Epidemiology and Biostatistics, Ministry of Education Key Laboratory of Environment and Health, School of Public Health, Tongji Medical College, Huazhong University of Science and Technology, Wuhan, China; Shanghai Jiao Tong University Affiliated Sixth People’s Hospital, CHINA

## Abstract

**Background:**

The influence of overall lifestyle behaviors on diabetic microvascular complications remains unknown. In addition, the potential mediating biomarkers underlying the association is unclear. This study aimed to examine the associations of the combined lifestyle factors with risks of total and individual microvascular complications among patients with type 2 diabetes (T2D) and to explore the potential mediation effects of metabolic biomarkers.

**Methods and findings:**

This retrospective cohort study included 15,104 patients with T2D free of macro- and microvascular complications at baseline (2006 to 2010) from the UK Biobank. Healthy lifestyle behaviors included noncurrent smoking, recommended waist circumference, regular physical activity, healthy diet, and moderate alcohol drinking. Outcomes were ascertained using electronic health records. Over a median of 8.1 years of follow-up, 1,296 cases of the composite microvascular complications occurred, including 558 diabetic retinopathy, 625 diabetic kidney disease, and 315 diabetic neuropathy, with some patients having 2 or 3 microvascular complications simultaneously. After multivariable adjustment for sociodemographic characteristics, history of hypertension, glycemic control, and medication histories, the hazard ratios (95% confidence intervals (CIs)) for the participants adhering 4 to 5 low-risk lifestyle behaviors versus 0 to 1 were 0.65 (0.46, 0.91) for diabetic retinopathy, 0.43 (0.30, 0.61) for diabetic kidney disease, 0.46 (0.29, 0.74) for diabetic neuropathy, and 0.54 (0.43, 0.68) for the composite outcome (all *Ps*-trend ≤0.01). Further, the population-attributable fraction (95% CIs) of diabetic microvascular complications for poor adherence to the overall healthy lifestyle (<4 low-risk factors) ranged from 25.3% (10.0%, 39.4%) to 39.0% (17.7%, 56.8%). In addition, albumin, HDL-C, triglycerides, apolipoprotein A, C-reactive protein, and HbA_1c_ collectively explained 23.20% (12.70%, 38.50%) of the associations between overall lifestyle behaviors and total diabetic microvascular complications. The key limitation of the current analysis was the potential underreporting of microvascular complications because the cases were identified via electronic health records.

**Conclusions:**

Adherence to overall healthy lifestyle behaviors was associated with a significantly lower risk of microvascular complications in patients with T2D, and the favorable associations were partially mediated through improving biomarkers of glycemic control, systemic inflammation, liver function, and lipid profile.

## Abstract 2

Tingting Geng and colleagues investigate whether lifestyle behaviours could affect microvascular complications among patients with type 2 diabetes in the UK Biobank.

## Author summary

**Why was this study done?:**

- Diabetic microvascular complications including diabetic retinopathy, diabetic neuropathy, and diabetic kidney disease have placed a significant disease burden.
- This study asked whether multiple lifestyle behaviors including waist circumference, smoking status, habitual diet, physical activity, and alcohol intake could jointly affect the microvascular complications, and whether the metabolic biomarkers could mediate the association.

**What did the researchers do and find?:**

- In this retrospective study of 15,104 patients with type 2 diabetes, participants adhering 4 to 5 low-risk lifestyle behaviors versus 0 to 1 had a 35% lower risk of diabetic retinopathy, 57% lower risk of diabetic kidney disease, 54% lower risk of diabetic neuropathy, and 46% lower risk of the composite outcome.
- Our study found that albumin, HDL-C, triglycerides, apolipoprotein A, C-reactive protein, and HbA_1c_ collectively explained 23.20% of the associations between overall lifestyle behaviors and diabetic microvascular complications.

**What do these findings mean?:**

- Our findings support the importance of public health programs and interventions targeting at improving health behaviors in combination to ameliorate the risk of diabetic microvascular complications.

## Introduction

Diabetes is a global public health crisis affecting greater than 0.5 billion adults worldwide [1]. Diabetic microvascular complications including diabetic retinopathy, diabetic neuropathy, and diabetic kidney disease have placed a significant health and economic burden borne by individuals, families, and health systems [2,3]. For example, diabetic retinopathy, the leading cause of vision loss, is present in nearly 30% of patients with diabetes [4]. Furthermore, both diabetic kidney disease and diabetic neuropathy may develop in approximately 50% of patients with diabetes [5,6]. Therefore, it is paramount to identify cost-effective strategies to prevent and delay the development of microvascular complications in patients with diabetes.

Beyond the glucose control by medications, the American Diabetes Association guideline has highlighted that both caregivers and patients should focus on how to optimize lifestyle behaviors to improve diabetes care [7]. Although lifestyle behaviors that are generally recommended, e.g., normal weight, no smoking, moderate alcohol drinking, healthy diet, and physically active, have been associated with lower risks of microvascular complications [8–14], to our best knowledge, the magnitudes of the joint association of multiple lifestyle factors with the development of microvascular complications in diabetes have not yet been quantified, which may have substantial public health implications on translating epidemiological findings to meaningful public health actions. In addition, several studies have linked lifestyle behaviors with a range of intermediate variables including lipid profile [15,16], liver function biomarkers [15,17–19], renal function biomarkers [20,21], blood pressure indices [22], glucose metabolism measures [23], and systemic inflammatory factors [15,16]; however, whether and the extent to which these metabolic biomarkers could mediate the association between lifestyle behaviors and diabetic microvascular complications remains unclear.

To shed light on the potential favorable association of overall lifestyle behaviors on microvascular complications in patients with diabetes, we examined the joint association of multiple lifestyle behaviors, including waist circumference (WC), smoking status, habitual diet, physical activity, and alcohol intake with risks of total microvascular complications, diabetic retinopathy, diabetic neuropathy, and diabetic kidney disease among patients with type 2 diabetes (T2D) who participated in the UK Biobank study. In addition, we also comprehensively evaluated the effect of a series of blood biomarkers on mediating the relationship between lifestyle behaviors and diabetic microvascular complications.

## Methods

### Study population

The UK Biobank is a large community-based prospective cohort study for common diseases of middle and older adults including over 500,000 participants aged 37 to 73 years from 22 sites across England, Scotland, and Wales between March 2006 and October 2010. Extensive data were obtained through touchscreen questionnaires, physical measurements, and biological samples at recruitment. Specific methods of data collection have been described previously [24,25].

Our sample of 15,104 was generated by including patients with T2D identified by using the algorithms method developed by the UK Biobank study [26] and excluding participants with prevalent macro- or microvascular complication cases, had incomplete information on lifestyle behaviors, or withdrawal from the study. The flowchart of patients included in the current study is present in **S1 Fig**.

The study was approved by the North West Multi-Centre Research Ethics Committee, the National Information Governance Board for Health and Social Care in England and Wales, and the Community Health Index Advisory Group in Scotland. All participants provided written informed consent. In the current analysis, we employed the UK Biobank study to test a priori hypothesis; we did not publish an analysis plan before conducting analyses between January 2022 and March 2022. The associations between lifestyle factors and the risk of microvascular complications in participants without excluding those with macrovascular complications and stratified analysis by preexisting cardiovascular disease (CVD) status were performed in response to peer review in July 2022. This study is reported as per the Strengthening the Reporting of Observational Studies in Epidemiology (STROBE) guideline (**S1 Checklist**).

### Measurements of lifestyle behaviors

Five lifestyle behaviors, namely, WC, smoking status, physical activity, habitual diet, and alcohol intake, were evaluated in the current analysis. We used WC instead of body mass index (BMI) to avoid the potential obesity paradox [27,28] as evidence found an obesity paradox when obesity was measured by BMI but not when measured by WC in patients with diabetes [29]. WC was measured using the Wessex nonstretchable sprung tape measurement, and low-risk WC was defined as <80 cm for women and <94 cm for men [30,31]. Data on smoking status were self-reported, and noncurrent smoking was defined as low-risk behavior. The frequency of all types of alcohol intake was reported using 6 predefined categories, between never to daily or almost daily. For participants who reported to drink alcohol, data on the average monthly or weekly alcohol intake from 6 types of alcohol beverages were collected. We calculated the average units of alcohol intake using the abovementioned information and defined low-risk drinking as moderate drinking (1 to 14 g/day for women or 1 to 28 g/day for men). Data on the type and duration of physical activity were derived from the questionnaire. Leisure-time physical activity score based on the 5 activities undertaken in the last 4 weeks was computed by multiplying the metabolic equivalent of task [MET] score of each activity by the minutes performed [32,33]. Light DIY (do-it-yourself), walking for pleasure, other exercises (e.g., swimming, cycling, keep fit, bowling), heavy DIY, and strenuous sports were given 1.5, 3.5, 4.0, 5.5, and 8.0 METs, respectively [34]. The midpoints of the frequency and duration of physical activities were used to calculate the time spent on each activity. We then classified the top third of the physical activity score as the low-risk group. In addition, we generated a dietary score to reflect the overall diet quality including 10 components, namely, fruits, vegetables, whole grains, fish, dairy, vegetable oils, refined grains, processed meat, unprocessed meat, and sugar-sweetened beverages. Low-risk diet was defined as meeting 5 or more ideal diet components [35]. Participants with each low-risk behavior were assigned 1 point; otherwise, 0 points. The overall healthy lifestyle score was the sum of individual score of the 5 lifestyle behaviors, ranging from 0 to 5, with higher score indicating healthier lifestyle.

### Assessment of the circulating biomarkers

Blood samples were collected from consenting participants at recruitment, separated by components and stored at UK Biobank (−80°C and LN_2_) until analysis. Blood biomarkers were externally validated with stringent quality control in the UK Biobank; full details on assay performance have been given elsewhere [36]. We selected the potential biological biomarkers mediating the association between lifestyle factors and microvascular complications based on knowledge of potential pathways, including glycemic control determined by glycated hemoglobin (HbA_1c_), lipid profile (total cholesterol [TC], high-density lipoprotein cholesterol [HDL-C], low-density lipoprotein cholesterol [LDL-C], triglycerides, apolipoprotein A, apolipoprotein B, and lipoprotein A), liver function (alanine aminotransferase [ALT], alkaline phosphatase [ALP], aspartate aminotransferase [AST], gamma glutamyltransferase [GGT], total bilirubin, total protein, and albumin), renal function (cystatin C, creatinine, urate, and urea), inflammation (C-reactive protein [CRP], and white blood cell count), and blood pressure indices (systolic blood pressure [SBP] and diastolic blood pressure [DBP]).

### Ascertainment of outcomes

Diabetic retinopathy (ICD-10: E113, E143, H280, H360), diabetic neuropathy (ICD-10: E114, E144, G590, G629, G632, G990), and diabetic kidney disease (ICD-10: E112, E142, N180, N181, N182, N183, N184, N185, N188, N189) cases were identified through linking the cohort database with the hospital inpatient admissions and death registries.

### Statistical analysis

Comparisons of baseline characteristics across the categories of the overall healthy lifestyle score were made using ANOVA or chi-squared test. We also compared the differences between patients included in the current analysis and those who were excluded due to missing values. Person-years were calculated from the date of recruitment to the date of death, first endpoint, lost to follow-up, or the end of follow-up, whichever came first. The lost to follow-up variable in the UK Biobank has been created by amalgamating data from 5 possible sources: (1) Death reported to UK Biobank by a relative; (2) NHS records indicate they are lost to follow-up; (3) NHS records indicate they have left the UK; (4) UK Biobank sources report they have left the UK; (5) Participant has withdrawn consent for future linkage. The end of follow-up dates were 1 April 2017, 17 September 2016, and 1 November 2016, for centers in England, Wales, and Scotland, respectively. Cox proportional hazards regression models were used to calculated hazard ratios (HRs) and 95% confidence intervals (CIs) for the associations of individual lifestyle behaviors and overall healthy lifestyle score with risks of total and individual microvascular complications in patients with T2D. We imputed the missing values of covariates (≤7%) using multiple imputations by chained equations with 5 imputations (SAS PROC MI with a fully conditional specification method and PROC MIANALYZE). Linear regression model and logistic regression model with all the covariates in the fully adjusted model were used to impute continuous variables and categorical variables, respectively. The percentage of missing values are present in **S1 Table**.

Three models were built. In Model 1, we adjusted for age (continuous, years), sex (male, female), Townsend Deprivation Index (continuous), and race/ethnicity (White, others). In Model 2, we further adjusted for education attainment (college or university degree, A/AS levels or equivalent or O levels/GCSEs, NVQ or HND or HNC or equivalent or other professional qualifications, none of the above), sleep duration (<6, 6 to 8, or ≥9 hours/day), family history of CVD (yes, no), family history of hypertension (yes, no), and prevalence of hypertension (yes, no). Finally, in Model 3, diabetes duration (continuous, years), HbA_1c_ (continuous, mmol/mol), use of diabetes medication (none, only oral medicine, insulin, and others), use of antihypertensive medication (yes, no), use of lipid-lowing medication (yes, no), and use of aspirin (yes, no) were additionally adjusted. Further, restricted cubic spline analysis was applied to test dose–response relationships between the healthy lifestyle score and risks of outcomes. We also calculated the population-attributable fractions (PAFs) using the %par SAS Macro (https://www.hsph.harvard.edu/donna-spiegelman/software/par/) to estimate the proportion of microvascular complications that could theoretically be avoided if all participants adhered to 4 or more low-risk lifestyle behaviors.

Mediation effects of biomarkers on the associations of overall lifestyle score with risks of total and individual microvascular complications were evaluated using mediation package in R. Indirect, direct, and total effects for each mediator were computed via combining the mediator and outcome models with the adjustment of all the covariates in Model 3. Nonparametric bootstrap resampling was used to compute the CIs of the proportions of mediations. We selected the available biomarkers from the UK Biobank for the mediation analyses based on knowledge of potential causal pathways to predisposing to microvascular complications or mortality [19,37–40]. The selected biomarkers were considered as potential mediators following two-step analysis. First, we assessed the associations of all biomarkers with the overall lifestyle score using the multivariable-adjusted linear regression models. Second, we evaluated the associations of biomarkers that were significantly associated with the overall lifestyle score, with risks of all the outcomes using the multivariable-adjusted Cox regression model. We then chose the biomarkers significantly associated with each outcome for the mediation analysis accordingly.

In addition, stratified analyses were conducted by age (≤60, >60 years), sex (female, male), education (less than college, college, or above), diabetes duration (≤3, >3 years), use of diabetes medication (yes, no), and HbA_1c_ (≤53, >53 mmol/mol). Interactions between the overall healthy lifestyle score and stratified factors on the risk of outcomes were examined using the likelihood ratio test by adding product terms in the multivariable-adjusted Cox models. Further, we examined the associations of different combinations of low-risk lifestyle behaviors with outcomes.

Several sensitivity analyses were conducted to test the robustness of our results. First, to minimize the potential reverse causation, we performed the analysis among patients with T2D after excluding the cases that occurred within 2 years of follow-up. Second, we generated the overall lifestyle score using low-risk drinking defined as moderate alcohol drinking and never drinking and repeated the main analysis using the new lifestyle score. Third, we constructed the healthy lifestyle score using BMI or waist-to-hip ratio instead of WC. Fourth, we generated a weighted healthy lifestyle score and examined the associations of the weighted healthy lifestyle score with risks of outcomes. Fifth, we investigated the association between the overall lifestyle score and risk of diabetic kidney disease, and mediation analysis for diabetic kidney disease with additional adjustment for kidney function biomarkers. Sixth, we performed the analysis via including the patients with CVD (*n =* 3,397) at baseline and stratified the associations by preexisting CVD status. Finally, given the potential competing risk of death highlighted during the peer review process, we assessed the associations of healthy lifestyle score with risks of microvascular complications using both the cause-specific hazard model and Fine and Gray subdistribution methods.

We used SAS V.9.4 and R software version 4.0.2 (R Foundation for Statistical Computing) for all statistical analyses. A two-tailed *P* < 0.05 was considered to be statistically significant.

## Results

### Baseline characteristics

Among 15,104 participants with T2D (60.3% male; mean age, 59.3 years), there were 3,406 (22.6%), 6,080 (40.3%), 4,062 (26.9%), 1,556 (10.3%) having 0 or 1, 2, 3, and 4 or 5 low-risk lifestyle behaviors, respectively. The baseline characteristics are shown in **Table 1**. Participants with more low-risk lifestyle behaviors were more likely to be men, White, less deprived, highly educated, sleep recommended hours, have a lower level of HbA_1c,_ and have a lower prevalence of hypertension. They were less likely to use aspirins and medications for diabetes, dyslipidemia, and hypertension. In addition, compared the participants who were excluded due to missing values, those included in the current analysis were more likely to be men, White, less deprived, highly educated, noncurrent smokers, physically active, moderate alcohol drinkers, and eat healthier (**S2 Table**).

**Table 1:** Baseline characteristics of the study population according to numbers of low-risk lifestyle behaviors.

	Numbers of low-risk lifestyle behaviors*					*P* value^†^
	Total	0–1	2	3	4–5	
Number of patients	15,104	3,406	6,080	4,062	1,556	-
Age, years	59.3 ± 7.1	58.4 ± 7.3	59.1 ± 7.1	60.0 ± 6.9	60.4 ± 6.7	<0.001
Men	9,112 (60.3)	1,820 (53.4)	3,573 (58.8)	2,624 (64.6)	1,095 (70.4)	<0.001
Ethnicity, White^‡^	13,207 (87.4)	2,822 (82.9)	5,346 (87.9)	3,634 (89.5)	1,405 (90.3)	<0.001
Townsend Deprivation Index^‡^	−0.6 ± 3.4	0.3 ± 3.5	−0.5 ± 3.4	−1.2 ± 3.2	−1.6 ± 3.0	<0.001
College or university degree^‡^	3,925 (26.0)	709 (20.8)	1,499 (24.7)	1,172 (28.9)	545 (35.0)	<0.001
Current smokers	1,593 (10.5)	1,046 (30.7)	434 (7.1)	103 (2.5)	10 (0.6)	<0.001
Healthy WC	2,164 (14.3)	74 (2.2)	376 (6.2)	786 (19.4)	928 (59.6)	<0.001
Physically active	5,043 (33.4)	102 (3.0)	1,051 (17.3)	2,512 (61.8)	1,378 (88.6)	<0.001
Moderate alcohol intake	9,875 (65.4)	502 (14.7)	4,373 (71.9)	3,510 (86.4)	1,490 (95.8)	<0.001
Healthy diet	3,282 (21.7)	53 (1.6)	714 (11.7)	1,419 (34.9)	1,096 (70.4)	<0.001
HbA_1c_, mmol/mol^‡^	51.9 ± 13.3	52.9 ± 14.0	52.3 ± 13.4	51.2 ± 12.9	49.6 ± 12.1	<0.001
Family history of CVD	8,484 (56.2)	1,850 (54.3)	3,421 (56.3)	2,342 (57.7)	871 (56.0)	0.04
Family history of hypertension	6,179 (40.9)	1,386 (40.7)	2,524 (41.5)	1,653 (40.7)	616 (39.6)	0.54
Prevalence of hypertension^‡^	10,041 (66.5)	2,367 (69.5)	4,169 (68.6)	2,629 (64.7)	876 (56.3)	<0.001
Diabetes duration, years^‡^	6.4 ± 9.0	6.6 ± 9.4	6.5 ± 9.2	6.1 ± 8.6	6.4 ± 8.4	0.07
Sleep duration (7–8 hours/day)^‡^	9,071 (60.1)	1,835 (53.9)	3,610 (59.4)	2,594 (63.9)	1,032 (66.3)	<0.001
Use of diabetes medication	10,318 (68.3)	2,396 (70.3)	4,239 (69.7)	2,705 (66.6)	978 (62.9)	<0.001
Use of antihypertensive medication	8,909 (59.0)	2,088 (61.3)	3,745 (61.6)	2,317 (57.0)	759 (48.8)	<0.001
Use of lipid-lowing medication	10,857 (71.9)	2,422 (71.1)	4,434 (72.9)	2,939 (72.4)	1,062 (68.3)	0.002
Use of aspirin	6,679 (44.2)	1,420 (41.7)	2,755 (45.3)	1,831 (45.1)	673 (43.3)	0.004

Data are presented as mean ± SD or n (%).

*Low-risk lifestyle behaviors: healthy WC (<94 cm for men, or <80 cm for women), noncurrent smokers, physically active (top third of total physical activity score), healthy diet (≥5 dietary components at ideal levels), and moderate alcohol intake (1–28 g/day for men; 1–14 g/day for women).

^**†**^*P* values for differences in baseline characteristics were estimated by ANOVA or chi-squared test.

^‡^The missing values of these covariates ranged from 0.1% to 7.0%.

CVD, cardiovascular disease; WC, waist circumference.

### Lifestyle behaviors and outcomes

**Fig 1:**
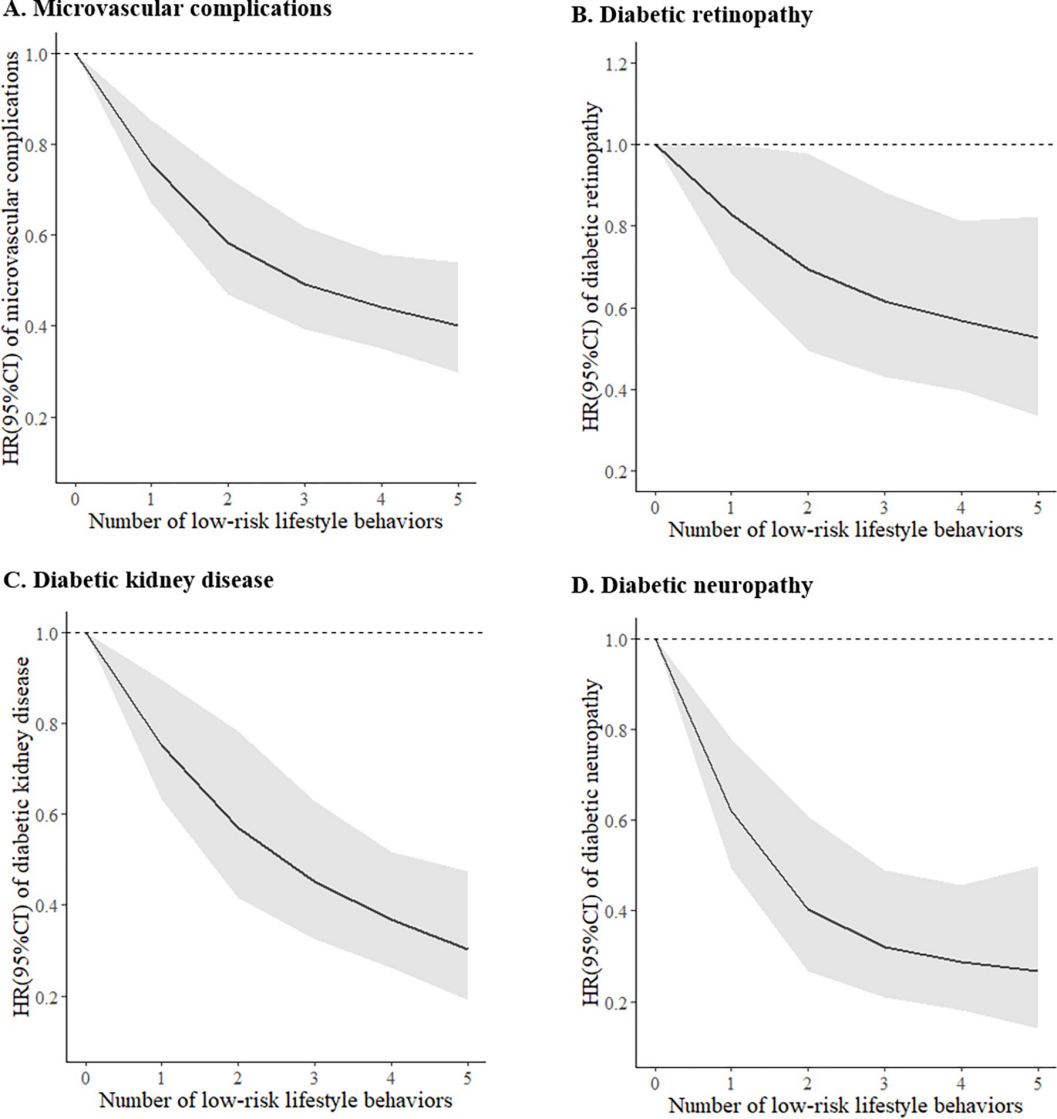
Dose–response relationship of the healthy lifestyle score with risk of microvascular complications among individuals with T2D. X-axis showed the numbers of low-risk lifestyle behaviors, and y-axis showed the HRs of the composite microvascular complications (**A**), diabetic retinopathy (**B**), diabetic kidney disease (**C**), and diabetic neuropathy (**D**). Black curves were HRs, and grey zones were 95% CIs. Multivariable-adjusted models were adjusted for age (continuous, years), sex (male, female), ethnicity (White, others), education attainment (college or university degree, A/AS levels or equivalent or O levels/GCSEs or equivalent or other professional qualifications, or none of the above), Townsend Deprivation Index (continuous), sleep duration (<6, 6–8, or ≥9 hours/day), family history of CVD (yes, no), family history of hypertension (yes, no), prevalence of hypertension (yes, no), diabetes duration (continuous, years), HbA_1c_ (continuous, mmol/mol), use of diabetes medication (none, only oral medication pills, or insulin or others), use of antihypertensive medication (yes, no), use of lipid-lowing medication (yes, no), and use of aspirin (yes, no). All *P*_-nonlinearity_ were ≥0.09 and all *P* for overall association were <0.001 (except for diabetic retinopathy: *P* for overall association = 0.008). CI, confidence interval; CVD, cardiovascular disease; HR, hazard ratio; T2D, type 2 diabetes.

During 117,445 person-years of follow-up (median 8.1 years; interquartile range 7.3 to 8.8 years; maximum 11.9 years), there occurred 1,639 (10.9%) deaths and 1,296 (8.6%) composite microvascular complications cases, including 558 (3.7%) diabetic retinopathy, 625 (4.1%) diabetic kidney disease, and 315 (2.1%) diabetic neuropathy. Among all the cases, one case of diabetic kidney disease was uniquely identified from death records. **S3 Table** shows the associations between individual lifestyle behaviors and all the outcomes. Being physically active, with lower WC, and moderate alcohol intake were associated with a lower risk of microvascular complications, while noncurrent smoking and healthy diet were not. The overall healthy lifestyle score was associated with lower risks of all the outcomes in a dose–response manner (all *Ps* for linear trend **≤0.01**; **Table 2 and Figs 1 and S2**). Compared with participants with 0 to 1 low-risk lifestyle behavior, participants with 4 to 5 low-risk lifestyle behaviors had HRs (95% CIs) of 0.65 (0.46, 0.91) for diabetic retinopathy, 0.43 (0.30, 0.61) for diabetic kidney disease, 0.46 (0.29, 0.74) for diabetic neuropathy, and 0.54 (0.43, 0.68) for the composite microvascular complications, respectively. For each number increment in low-risk lifestyle behavior, there was a 13% lower risk of diabetic retinopathy (HR, 0.87; 95% CI: 0.80, 0.95), 22% lower risk of diabetic kidney disease (HR, 0.78; 95% CI: 0.72, 0.85), 27% lower risk of diabetic neuropathy (HR, 0.73; 95% CI: 0.65, 0.83), and a 18% lower risk of the composite microvascular complications (HR, 0.82; 95% CI: 0.77, 0.87).

**Table 2:** HRs (95% CIs) of microvascular complications according to the overall lifestyle behaviors among individuals with T2D.

	Numbers of low-risk lifestyle behaviors					
	0–1	2	3	4–5	*P*_trend_	HR _continuous_
**Composite microvascular complications**						
Cases/Person-years*	378/25,684	507/47,476	312/31,985	99/12,301	-	-
Unadjusted	1.00	0.71 (0.67, 0.76)	0.65 (0.61, 0.69)	0.54 (0.48, 0.59)	<0.001	0.82 (0.80, 0.84)
Model 1	1.00	0.70 (0.62, 0.80)	0.62 (0.53, 0.72)	0.51 (0.40, 0.63)	<0.001	0.80 (0.76, 0.85)
Model 2	1.00	0.71 (0.62, 0.81)	0.63 (0.54, 0.74)	0.53 (0.42, 0.67)	<0.001	0.81 (0.77, 0.86)
Model 3	1.00	0.71 (0.62, 0.81)	0.65 (0.56, 0.76)	0.54 (0.43, 0.68)	<0.001	0.82 (0.77, 0.87)
PAF^†^, %	-	-	-	25.3 (10.0, 39.4)	-	-
**Diabetic retinopathy**						
Cases/Person-years*	149/26,283	224/48,250	139/32,442	46/12,439	-	-
Unadjusted	1.00	0.81 (0.74, 0.89)	0.75 (0.67, 0.83)	0.64 (0.56, 0.75)	<0.001	0.87 (0.84, 0.91)
Model 1	1.00	0.82 (0.66, 1.01)	0.74 (0.58, 0.93)	0.63 (0.45, 0.88)	0.002	0.87 (0.79, 0.94)
Model 2	1.00	0.82 (0.66, 1.01)	0.74 (0.59, 0.94)	0.64 (0.45, 0.89)	0.003	0.87 (0.79, 0.95)
Model 3	1.00	0.82 (0.66, 1.01)	0.76 (0.60, 0.96)	0.65 (0.46, 0.91)	0.01	0.87 (0.80, 0.95)
PAF^†^, %	-	-	-	20.3 (−3.7, 42.1)	-	-
**Diabetic kidney disease**						
Cases/Person-years*	188/26,360	245/48,401	153/32,554	39/12,542	-	-
Unadjusted	1.00	0.69 (0.64, 0.76)	0.64 (0.58, 0.71)	0.42 (0.36, 0.49)	<0.001	0.78 (0.76, 0.81)
Model 1	1.00	0.67 (0.55, 0.81)	0.58 (0.47, 0.73)	0.38 (0.27, 0.54)	<0.001	0.75 (0.69, 0.82)
Model 2	1.00	0.69 (0.57, 0.83)	0.61 (0.49, 0.77)	0.41 (0.29, 0.59)	<0.001	0.77 (0.71, 0.84)
Model 3	1.00	0.69 (0.57, 0.84)	0.63 (0.51, 0.79)	0.43 (0.30, 0.61)	<0.001	0.78 (0.72, 0.85)
PAF^†^, %	-	-	-	39.0 (17.7, 56.8)	-	-
**Diabetic neuropathy**						
Cases/Person-years*	110/26,409	122/48,553	61/32,742	22/12,533	-	-
Unadjusted	1.00	0.60 (0.53, 0.67)	0.44 (0.38, 0.51)	0.42 (0.34, 0.51)	<0.001	0.71 (0.68, 0.75)
Model 1	1.00	0.59 (0.46, 0.77)	0.43 (0.32, 0.60)	0.41 (0.26, 0.65)	<0.001	0.71 (0.63, 0.80)
Model 2	1.00	0.61 (0.47, 0.79)	0.46 (0.34, 0.64)	0.48 (0.30, 0.76)	<0.001	0.74 (0.65, 0.83)
Model 3	1.00	0.61 (0.47, 0.79)	0.47 (0.34, 0.64)	0.46 (0.29, 0.74)	<0.001	0.73 (0.65, 0.83)
PAF^†^, %	-	-	-	26.4 (−6.5, 54.1)	-	-

**Model 1:** age (continuous, years), sex (male, female), Townsend Deprivation Index (continuous), and race/ethnicity (White, others).

**Model 2: Model 1** + education attainment (college or university degree, A/AS levels or equivalent or O levels/GCSEs or equivalent or other professional qualifications, or none of the above), sleep duration (<6, 6–8, or ≥9 hours/day), family history of CVD (yes, no), family history of hypertension (yes, no), and prevalence of hypertension (yes, no).

**Model 3: Model 2** + diabetes duration (continuous, years), HbA_1c_ (continuous, mmol/mol), use of diabetes medication (none, only oral medication pills, or insulin or others), use of antihypertensive medication (yes, no), use of lipid-lowing medication (yes, no), and use of aspirin (yes, no).

*The composite microvascular complications were defined as the occurrence of any types of microvascular complications including diabetic retinopathy, diabetic kidney disease, and diabetic neuropathy. The person-years for the composite microvascular complications were calculated from the date of recruitment to the date of death, diagnosis of any types of microvascular complications, lost to follow-up, or the end of follow-up, whichever came first. The person-years for each outcome were calculated individually without censoring other types of microvascular complications.

^†^PAFs based on Model 3 were calculated to theoretically estimate the proportion of each outcome in this study population that could have been prevented if the population had ≥4 low-risk lifestyle behaviors.

CI, confidence interval; CVD, cardiovascular disease; HR, hazard ratio; PAF, population-attributable fraction; T2D, type 2 diabetes.

In addition, the estimated PAFs of nonadherence to 4 or more low-risk lifestyle factors were 39.0% (17.7%, 56.8%) for diabetic kidney disease and 25.3% (10.0%, 39.4%) for the composite microvascular complications (**Table 2**).

### Mediation analysis

All the biomarkers were significantly associated with the overall lifestyle score except for total protein, lipoprotein A, and SBP (**S4 Table**). The associations between the selected biomarkers and all outcomes are shown in **S5 Table**. Six significant mediators were detected on the associations of lifestyle score with risk of the composite microvascular complications and diabetic kidney disease, namely, albumin, HDL-C, triglycerides, apolipoprotein A, CRP, and HbA_1c_. The relationship between the lifestyle behaviors and risk of diabetic neuropathy was mediated by cystatin C, GGT, total bilirubin, albumin, HDL-C, triglycerides, apolipoprotein A, CRP, and HbA_1c_ with the proportion of mediation effect ranging from 3.22% to 11.35%. Collectively, the mediators explained 23.20%, 24.40%, and 31.90% of the associations of overall lifestyle behaviors with composite microvascular complications, diabetic kidney disease, and diabetic neuropathy, respectively. In addition, our data showed that among all the potential biomarkers, only HbA_1c_ was a significant mediator that explained 15.26% of the relationship between the overall lifestyle score and risk of diabetic retinopathy (**Table 3**).

**Table 3:** Association of healthy lifestyle scores with microvascular complications mediated by biomarkers among individuals with T2D*.

	Total effect				Natural direct effect				Natural indirect effect				Proportion eliminated	
	Beta	Lower	Upper	*P*	Beta	Lower	Upper	*P*	Beta	Lower	Upper	*P*	% (95%CI)^†^	*P*
**Model 1**														
***Microvascular complications***														
Albumin (g/L)	−0.0152	−0.0236	−0.0080	<0.001	−0.0130	−0.0214	−0.0062	<0.001	−0.0022	−0.0029	−0.0015	<0.001	14.17 (9.06, 24.52)	<0.001
HDL-C (mmol/L)	−0.0156	−0.0228	−0.0086	<0.001	−0.0145	−0.0217	−0.0077	<0.001	−0.0011	−0.0017	−0.0005	<0.001	6.89 (3.07, 13.43)	<0.001
Triglycerides (mmol/L)	−0.0191	−0.0271	−0.0118	<0.001	−0.0179	−0.0260	−0.0107	<0.001	−0.0012	−0.0020	−0.0004	<0.001	6.11 (2.26, 12.12)	<0.001
Apolipoprotein A (g/L)	−0.0155	−0.0232	−0.0087	<0.001	−0.0150	−0.0226	−0.0081	<0.001	−0.0005	−0.0010	−0.0001	0.01	3.22 (0.65, 7.26)	0.01
CRP (mg/L)	−0.0190	−0.0271	−0.0116	<0.001	−0.0166	−0.0248	−0.0095	<0.001	−0.0023	−0.0037	−0.0009	0.002	12.34 (4.65, 22.24)	0.002
HbA_1c_ (mmol/mol)	−0.0180	−0.0259	−0.0112	<0.001	−0.0156	−0.0234	−0.0089	<0.001	−0.0024	−0.0032	−0.0018	<0.001	13.24 (8.55, 21.17)	<0.001
Total mediation	-	-	-	-	-	-	-	-	-	-	-	-	27.20 (15.10, 44.00)	<0.001
***Diabetic retinopathy***														
HbA_1c_ (mmol/mol)	−0.0049	−0.0105	−0.0010	0.01	−0.0035	−0.0087	0.0002	0.08	−0.0014	−0.0020	−0.0010	<0.001	29.22 (14.76, 116.27)	0.01
Total mediation	-	-	-	-	-	-	-	-	-	-	-	-	29.22 (14.76, 116.27)	0.01
***Diabetic kidney disease***														
Albumin (g/L)	−0.0101	−0.0166	−0.0047	<0.001	−0.0085	−0.0148	−0.0032	<0.001	−0.0016	−0.0022	−0.0011	<0.001	15.79 (10.00, 32.11)	<0.001
HDL-C (mmol/L)	−0.0106	−0.0174	−0.0051	<0.001	−0.0098	−0.0164	−0.0043	<0.001	−0.0008	−0.0013	−0.0004	<0.001	7.70 (3.44, 15.84)	<0.001
Triglycerides (mmol/L)	−0.0120	−0.0193	−0.0067	<0.001	−0.0111	−0.0184	−0.0057	<0.001	−0.0009	−0.0016	−0.0003	0.004	7.57 (2.53, 16.15)	0.004
Apolipoprotein A (g/L)	−0.0105	−0.0172	−0.0051	<0.001	−0.0102	−0.0169	−0.0050	<0.001	−0.0003	−0.0007	−0.00001	0.04	3.01 (0.08, 6.95)	0.04
CRP (mg/L)	−0.0122	−0.0188	−0.0065	<0.001	−0.0099	−0.0166	−0.0044	<0.001	−0.0023	−0.0034	−0.0012	<0.001	18.81 (9.02, 35.49)	<0.001
HbA_1c_ (mmol/mol)	−0.0124	−0.0195	−0.0065	<0.001	−0.0117	−0.0188	−0.0058	<0.001	−0.0008	−0.0012	−0.0004	<0.001	6.29 (3.26, 12.44)	<0.001
Total mediation	-	-	-	-	-	-	-	-	-	-	-	-	29.00 (14.90, 48.80)	<0.001
***Diabetic neuropathy***														
Cystatin C (mg/L)	−0.0109	−0.0180	−0.0056	<0.001	−0.0095	−0.0164	−0.0041	<0.001	−0.0014	−0.0020	−0.0009	<0.001	12.79 (7.12, 23.30)	<0.001
GGT (U/L)	−0.0111	−0.0183	−0.0058	<0.001	−0.0100	−0.0172	−0.0048	<0.001	−0.0011	−0.0017	−0.0004	<0.001	9.73 (3.37, 20.35)	<0.001
Total bilirubin, μmol/L	−0.0112	−0.0185	−0.0058	<0.001	−0.0106	−0.0181	−0.0053	<0.001	−0.0006	−0.0012	−0.0002	0.01	5.38 (1.22, 11.67)	0.01
Albumin (g/L)	−0.0113	−0.0189	−0.0056	<0.001	−0.0105	−0.0178	−0.0049	<0.001	−0.0008	−0.0013	−0.0004	<0.001	7.22 (3.82, 12.91)	<0.001
HDL-C (mmol/L)	−0.0116	−0.0196	−0.0057	<0.001	−0.0109	−0.0186	−0.0052	<0.001	−0.0007	−0.0012	−0.0003	<0.001	6.33 (2.84, 12.35)	<0.001
Triglycerides (mmol/L)	−0.0115	−0.0183	−0.0058	<0.001	−0.0104	−0.0172	−0.0049	<0.001	−0.0012	−0.0018	−0.0006	<0.001	10.00 (4.99, 17.74)	<0.001
Apolipoprotein A (g/L)	−0.0115	−0.0193	−0.0058	<0.001	−0.0111	−0.0188	−0.0054	<0.001	−0.0004	−0.0008	−0.0001	0.002	3.62 (1.10, 7.24)	0.002
CRP (mg/L)	−0.0114	−0.0194	−0.0064	<0.001	−0.0098	−0.0173	−0.0048	<0.001	−0.0017	−0.0027	−0.0008	<0.001	14.48 (6.30, 26.18)	<0.001
HbA_1c_ (mmol/mol)	−0.0099	−0.0168	−0.0043	<0.001	−0.0088	−0.0154	−0.0035	<0.001	−0.0010	−0.0015	−0.0006	<0.001	10.32 (6.43, 19.42)	<0.001
Total mediation	-	-	-	-	-	-	-	-	-	-	-	-	38.40 (21.50, 58.70)	<0.001
**Model 2**														
***Microvascular complications***														
Albumin (g/L)	−0.0128	−0.0208	−0.0063	<0.001	−0.0116	−0.0193	−0.0052	<0.001	−0.0012	−0.0018	−0.0008	<0.001	9.63 (5.30, 19.36)	<0.001
HDL-C (mmol/L)	−0.0128	−0.0200	−0.0062	<0.001	−0.0117	−0.0187	−0.0053	<0.001	−0.0011	−0.0018	−0.0005	<0.001	8.48 (3.83, 17.62)	<0.001
Triglycerides (mmol/L)	−0.0167	−0.0245	−0.0096	<0.001	−0.0155	−0.0236	−0.0086	<0.001	−0.0012	−0.0020	−0.0004	<0.001	7.16 (2.37, 14.21)	<0.001
Apolipoprotein A (g/L)	−0.0128	−0.0206	−0.0064	<0.001	−0.0122	−0.0199	−0.0056	<0.001	−0.0006	−0.0011	−0.0001	0.01	4.44 (0.99, 10.73)	0.01
CRP (mg/L)	−0.0168	−0.0247	−0.0099	<0.001	−0.0150	−0.0228	−0.0081	<0.001	−0.0018	−0.0029	−0.0006	0.002	10.69 (3.64, 20.34)	0.002
HbA_1c_ (mmol/mol)	−0.0174	−0.0256	−0.0106	<0.001	−0.0161	−0.0241	−0.0094	<0.001	−0.0013	−0.0020	−0.0008	<0.001	7.72 (4.55, 13.24)	<0.001
Total mediation	-	-	-	-	-	-	-	-	-	-	-	-	23.20 (12.70, 38.50)	<0.001
***Diabetic retinopathy***														
HbA_1c_ (mmol/mol)	−0.0050	−0.0104	−0.0012	0.004	−0.0043	−0.0094	−0.0005	0.02	−0.0008	−0.0011	−0.0005	<0.001	15.26 (7.26, 54.39)	0.004
Total mediation	-	-	-	-	-	-	-	-	-	-	-	-	15.26 (7.26, 54.39)	0.004
***Diabetic kidney disease***														
Albumin (g/L)	−0.0098	−0.0167	−0.0041	<0.001	−0.0087	−0.0154	−0.0007	<0.001	−0.0011	−0.0016	−0.0007	<0.001	11.32 (6.69, 24.54)	<0.001
HDL-C (mmol/L)	−0.0102	−0.0170	−0.0046	<0.001	−0.0092	−0.0160	−0.0037	<0.001	−0.0010	−0.0016	−0.0005	<0.001	9.94 (4.67, 21.00)	<0.001
Triglycerides (mmol/L)	−0.0117	−0.0190	−0.0062	<0.001	−0.0107	−0.0180	−0.0054	<0.001	−0.0010	−0.0016	−0.0003	0.004	8.14 (2.74, 17.23)	0.004
Apolipoprotein A (g/L)	−0.0101	−0.0167	−0.0046	<0.001	−0.0096	−0.0161	−0.0043	<0.001	−0.0005	−0.0009	−0.0001	0.01	4.90 (1.28, 10.74)	0.01
CRP (mg/L)	−0.0119	−0.0188	−0.0064	<0.001	−0.0099	−0.0167	−0.0046	<0.001	−0.0020	−0.0030	−0.0011	<0.001	16.94 (8.87, 31.46)	<0.001
HbA_1c_ (mmol/mol)	−0.0126	−0.0203	−0.0062	<0.001	−0.0121	−0.0198	−0.0058	<0.001	−0.0005	−0.0009	−0.0002	0.002	3.83 (1.16, 8.42)	0.002
Total mediation	-	-	-	-	-	-	-	-	-	-	-	-	24.40 (12.90, 41.20)	<0.001
***Diabetic neuropathy***														
Cystatin C (mg/L)	−0.0091	−0.0155	−0.0040	<0.001	−0.0081	−0.0142	−0.0031	<0.001	−0.0010	−0.0016	−0.0005	<0.001	11.35 (5.11, 23.19)	<0.001
GGT (U/L)	−0.0091	−0.0156	−0.0042	<0.001	−0.0083	−0.0147	−0.0034	<0.001	−0.0008	−0.0014	−0.0002	0.01	8.63 (1.79, 20.46)	0.01
Total bilirubin, μmol/L	−0.0094	−0.0161	−0.0045	<0.001	−0.0091	−0.0158	−0.0043	<0.001	−0.0003	−0.0006	−0.00004	0.01	3.22 (0.45, 7.65)	0.01
Albumin (g/L)	−0.0089	−0.0155	−0.0038	<0.001	−0.0085	−0.0151	−0.0035	<0.001	−0.0004	−0.0007	−0.0001	<0.001	4.65 (1.63, 10.41)	<0.001
HDL-C (mmol/L)	−0.0089	−0.0170	−0.0036	<0.001	−0.0083	−0.0163	−0.0032	<0.001	−0.0006	−0.0011	−0.0002	<0.001	6.57 (2.38, 15.74)	<0.001
Triglycerides (mmol/L)	−0.0093	−0.0157	−0.0043	<0.001	−0.0084	−0.0148	−0.0037	<0.001	−0.0009	−0.0016	−0.0004	<0.001	9.82 (4.45, 19.65)	<0.001
Apolipoprotein A (g/L)	−0.0087	−0.0153	−0.0035	<0.001	−0.0084	−0.0151	−0.0033	<0.001	−0.0004	−0.0007	−0.0001	0.02	4.07 (0.78, 10.09)	0.02
CRP (mg/L)	−0.0093	−0.0163	−0.0046	<0.001	−0.0083	−0.015	−0.0038	<0.001	−0.0010	−0.0018	−0.0003	<0.001	11.24 (3.87, 23.00)	<0.001
HbA_1c_ (mmol/mol)	−0.0089	−0.0153	−0.0037	<0.001	−0.0083	−0.0146	−0.0032	<0.001	−0.0006	−0.0009	−0.0003	<0.001	6.52 (3.34, 13.27)	<0.001
Total mediation	-	-	-	-	-	-	-	-	-	-	-	-	31.90 (16.40, 52.80)	<0.001

**Model 1:** unadjusted model.

**Model 2:** age (continuous, years), sex (male, female), ethnicity (White, others), education attainment (college or university degree, A/AS levels or equivalent or O levels/GCSEs or equivalent or other professional qualifications, or none of the above), Townsend Deprivation Index (continuous), sleep duration (<6, 6–8, or ≥9 hours/day), family history of CVD (yes, no), family history of hypertension (yes, no), prevalence of hypertension (yes, no), diabetes duration (continuous, years), use of diabetes medication (none, only oral medication pills, or insulin or others), HbA_1c_ (continuous, mmol/mol), use of antihypertensive medication (yes, no), use of lipid-lowing medication (yes, no), and use of aspirin (yes, no). HbA_1c_ (continuous, mmol/mol) levels were not adjusted when HbA_1c_ was analyzed as a mediator.

*The levels of biomarkers were nature log-transformed before analyses.

^†^1,000 bootstrap resampling.

CI, confidence interval; CRP, C-reactive protein; CVD, cardiovascular disease; HDL-C, high-density lipoprotein cholesterol; T2D, type 2 diabetes.

### Secondary analysis and sensitivity analysis

Consistent results were observed when analyses were stratified by age, sex, education, diabetes duration, use of hypoglycemic medication, and HbA_1c_ level. No significant interaction was observed between the healthy lifestyle score and the stratified factors on the outcomes considering multiple comparisons (**S3 Fig**). Further, the results of different combinations of low-risk lifestyle factors showed that the increased numbers of low-risk lifestyle factors were associated with graded lower risks of diabetic retinopathy, diabetic kidney disease, diabetic neuropathy, and the composite microvascular complications (**S6 Table**).

In the sensitivity analyses, the results were generally robust when excluding patients with events that occurred within the first 2 years of follow-up, defining low-risk alcohol intake as moderate drinking and nondrinking, generating the lifestyle score using BMI or waist-to-hip ratio instead of WC, or generating the overall lifestyle score as a weighted score (**S7–S10 Tables**). The association between overall lifestyle behaviors and risk of diabetic kidney disease was slightly attenuated when estimated glomerular filtration rate (eGFR) was additionally adjusted, and the results of mediation analysis for diabetic kidney disease were largely unchanged with the additional adjustment of eGFR (**S11 and S12 Tables**). Further, we observed similar results when patients with preexisting CVD were included and in patients with preexisting CVD, although diabetic retinopathy did not reach statistical significance in patients with preexisting CVD probably due to the insufficient power (**S13 and S14 Tables**). Finally, consistent results were demonstrated when we used 2 competing risk models accounting for the death (**S15 Table**).

## Discussion

In this retrospective cohort study of patients with T2D, adherence to a greater number of healthy lifestyle behaviors, including recommended WC, noncurrent smoking, physically active, healthy diet, and moderate alcohol drinking, was inversely associated with lower risks of diabetic retinopathy, diabetic kidney disease, diabetic neuropathy, and the composite microvascular complications. For each number increment in low-risk lifestyle behavior, there was an 18% lower risk of developing diabetic microvascular complications. Moreover, the results of PAFs suggested that 25.3% of the diabetic microvascular complications could have been avoided if the patients with T2D had 4 or more healthy lifestyle behaviors. In addition, the mediators collectively explained 23.20% of the associations between the overall healthy lifestyle score and diabetic microvascular complications. Specifically, CRP, albumin, HbA_1c_, and lipids profile (HDL-C, triglycerides, and apolipoprotein A) could explain 4.44% to 10.69% of the association between overall lifestyle behaviors and the total diabetic microvascular complications.

Our study contributes to the literature regarding the influence of combined healthy lifestyle behaviors on the risk of diabetic microvascular complications. To date, many studies have been performed to evaluate the relationship between individual lifestyle behaviors and risk of diabetic microvascular complications; however, the joint association of multiple lifestyle behaviors with microvascular complications remains unknown. For example, the Irish Longitudinal Study showed that a history of smoking was associated with a higher risk of developing microvascular complications [8]. The Ongoing Telmisartan Alone and in Combination with Ramipril Global Endpoint Trial (ONTARGET) studies demonstrated that adherence to a healthy dietary pattern (the Alternate Healthy Eating Index) [9], being physically active, and moderate alcohol consumption [12] were associated with a lower risk of incident chronic kidney disease among patients with T2D. Furthermore, general obesity and abdominal obesity were associated with higher risks of diabetic kidney disease [41], diabetic retinopathy [13], and diabetic neuropathy [42].

However, the results of lifestyle interventions on microvascular complications among patients with diabetes or impaired glucose tolerance in clinical trials were inconsistent. The Steno-2 randomized trial including 160 patients with T2D and persistent microalbuminuria showed pharmacological therapies in combination with lifestyle behavior modifications, including adopting a healthy diet, engaging regular physical activity, and participating in smoking cessation courses, significantly reduced the risk of diabetic nephropathy, retinopathy, and neuropathy [43]. Further, the China Da Qing Diabetes Prevention Study including 577 participants with impaired glucose tolerance reported that healthy diet and exercise interventions in combination resulted in a 47% reduction in the diabetic retinopathy incidence, but no beneficial effects were observed for diabetic nephropathy or neuropathy [44]. In addition, the Look AHEAD trial consisting of 5,145 overweight or obese patients with T2D, which focused on weight management through increased energy deficit and physical activity, resulted in a significant decrease in chronic kidney disease [45], but not diabetic neuropathy measured by physical examinations [46]. Notably, microvascular complications were not predefined primary outcomes in these trials and small numbers of cases might partially explained the heterogeneities in these findings (e.g., 296 cases of very-high-risk chronic kidney disease in the Look AHEAD trial). Further trials with proper designs are needed to corroborate our findings in the future.

Our mediation analyses contribute to better understanding the lower risk of microvascular complications associated with lifestyle behaviors. Our data showed that the associations of overall lifestyle behaviors with diabetic kidney disease, diabetic neuropathy, and total microvascular complications may be explained by the improvement in glycemic control, liver function, lipid profile, and systemic inflammation, with lifestyle behaviors related lower risk of diabetic neuropathy might be additionally explained by kidney function amelioration. However, our data showed that the association between lifestyle and diabetic retinopathy was mainly through the glycemic control rather than other pathways. Our results corroborate prior findings from the observational studies. For example, intensive lifestyle intervention including physical activity and healthy diet recommendations could benefit glycemic control [47]. Adherence to a combined healthy lifestyle score including healthy diet, physically active, nonsmoking, healthy sleep, and social support were associated with lower concentrations of inflammatory markers [48]. Chronic Renal Insufficiency Cohort (CRIC) Study showed that combined healthy lifestyle characterized as physically active, nonsmoking, and BMI ≥25 kg/m^2^ were associated with lower risks of atherosclerotic events and kidney function decline among patients with chronic kidney disease [20]. Furthermore, lifestyle modifications including promoting healthy diet, physical activity, and weight loss could significantly improve liver function, renal function, lipid profile, endothelial dysfunction, and reduce systemic inflammation in interventional studies [49–54].

The current study is among the first to investigate the relationship between the overall lifestyle behaviors and diabetic microvascular complications. The strengths of this study included the large sample size, long period of follow-up, and extensive collection of data on clinical biomarkers, which allowed us to comprehensively evaluate the potential mechanisms underlying the observed associations. Despite the strengths, this study should be interpreted in the light of its potential limitations. First, as the microvascular complications were identified via hospital inpatient records and death registries, there might be underreporting of the cases, for example, primary care data were not completely available currently. Second, the self-reported and one-time assessment of lifestyle behaviors data are susceptible to measurement errors. In addition, information on lifestyle behaviors was collected at recruitment and the behaviors may change over time; hence, the observed associations might be attenuated due to nondifferential misclassification bias. Third, mediation analysis assumes causality between lifestyles behaviors and biological biomarkers, although both the lifestyle behaviors and biological mediators were assessed at the same time in the UK Biobank. Future studies with repeatedly measured data are required to replicate our findings. Fourth, our study is limited in terms of ethnic diversity (>85% Whites); our results may not be directly generalized to other ethnic groups. Fifth, our study was based on a retrospective sampling from the UK Biobank study; hence, the causality should be interpreted with caution. Sixth, the UK Biobank is not representative of the general population of the UK, particularly relating to socioeconomic deprivation, lifestyles, and noncommunicable disease, with evidence of the healthy volunteer selection bias. Finally, residual or unknown confounding could not be excluded due to the observational study design, although we have in our effort to adjust for the potential confounding factors.

## Conclusions

Our findings suggested that adherence to overall healthy lifestyle behaviors, including abstaining from smoking, abdominal obesity management, adopting a healthy diet, engaging in regular physical activity, and moderate alcohol drinking, was associated with a lower risk of diabetic microvascular complications. Our findings support the importance of public health programs and interventions targeting at improving health behaviors in combination to ameliorate the risk of diabetic microvascular complications. In addition, our data showed significant mediation effect of biomarkers involving of glycemic control, liver function, lipid profile, and systemic inflammation on the associations between lifestyle behaviors and microvascular complications. Further studies are warranted to confirm our findings.

## Supporting information

S1 Checklist STROBE Statement—Checklist of items that should be included in reports of *cohort studies*. (DOCX)

S1 Fig Flowchart of the selection of the study population from the UK Biobank study. (TIF)

S2 Fig Dose–response relationship of the healthy lifestyle score with risk of microvascular complications among individuals with T2D (unadjusted model). X-axis showed the numbers of low-risk lifestyle behaviors, and y-axis showed the HRs of the composite microvascular complications (**A**), diabetic retinopathy (**B**), diabetic kidney disease (**C**), and diabetic neuropathy (**D**). Black curves were HRs, and grey zones were 95% CIs. All *P*_-nonlinearity_ were ≥0.10, and all *P* for overall association were <0.001 (except for diabetic retinopathy: *P* for overall association = 0.005). CI, confidence interval; HR, hazard ratio; T2D, type 2 diabetes.
(TIF)

S3 Fig Stratified analyses of the associations of overall healthy lifestyle score with microvascular complications. **Model 1:** unadjusted model. **Model 2:** age (continuous, years), sex (male, female), race (White, others), education attainment (college or university degree, A/AS levels or equivalent or O levels/GCSEs or equivalent or other professional qualifications, or none of the above), Townsend Deprivation Index (continuous), sleep duration (<6, 6–8, or ≥9 hours/day), family history of CVD (yes, no), family history of hypertension (yes, no), prevalence of hypertension (yes, no), diabetes duration (continuous, years), use of diabetes medication (none, only oral medication pills, or insulin or others), HbA_1c_ (continuous, mmol/mol), use of antihypertensive medication (yes, no), use of lipid-lowing medication (yes, no), and use of aspirin (yes, no), with exception of stratifying factors. Interactions were tested based on Model 2.
(TIF)

S1 Table Percentage of missing values of the covariates. (DOCX)

S2 Table Baseline characteristics between individuals with T2D included in the study and those excluded due to the missing values. (DOCX)

S3 Table HRs (95% CIs) of microvascular complications according to individual lifestyle behaviors in individuals with T2D. CI, confidence interval; HR, hazard ratio; T2D, type 2 diabetes.
(DOCX)

S4 Table Multivariable-adjusted linear regression models for the association between the healthy lifestyle score and biomarker levels among individuals with T2D. (DOCX)

S5 Table Risk estimates of microvascular complications associated with the selected biomarkers (1-SD increment) among individuals with T2D. (DOCX)

S6 Table HRs (95% CIs) of microvascular complications according to different combinations of low-risk lifestyle factors among individuals with T2D. CI, confidence interval; HR, hazard ratio; T2D, type 2 diabetes.
(DOCX)

S7 Table HRs (95% CIs) of microvascular complications according to the overall lifestyle score among individuals with T2D after excluding the cases that occurred within first 2 years of follow-up. CI, confidence interval; HR, hazard ratio; T2D, type 2 diabetes.
(DOCX)

S8 Table HRs (95% CIs) of microvascular complications according to the healthy lifestyle score using moderate drinking and nondrinking as the low-risk behavior in individuals with T2D. CI, confidence interval; HR, hazard ratio; T2D, type 2 diabetes.
(DOCX)

S9 Table HRs (95% CIs) of microvascular complications according to the healthy lifestyle score using optimal BMI or optimal WHR as the low-risk behavior in individuals with T2D. BMI, body mass index; CI, confidence interval; HR, hazard ratio; T2D, type 2 diabetes; WHR, waist-to-hip ratio.
(DOCX)

S10 Table HRs (95% CIs) of microvascular complications according to the weighted lifestyle score in individuals with T2D. CI, confidence interval; HR, hazard ratio; T2D, type 2 diabetes.
(DOCX)

S11 Table HRs (95% CIs) of diabetic kidney disease according to the healthy lifestyle score among individuals with T2D with additional adjustment for eGFR. CI, confidence interval; eGFR, estimated glomerular filtration rate; HR, hazard ratio; T2D, type 2 diabetes.
(DOCX)

S12 Table Possible mediators of the associations between the healthy lifestyle score and diabetic kidney disease among individuals with T2D with additional adjustment for eGFR. eGFR, estimated glomerular filtration rate; T2D, type 2 diabetes.
(DOCX)

S13 Table HRs (95% CIs) of microvascular complications according to the healthy lifestyle score in 18,501 individuals with T2D including patients with preexisting macrovascular disease. CI, confidence interval; HR, hazard ratio; T2D, type 2 diabetes.
(DOCX)

S14 Table HRs (95% CIs) of microvascular complications according to the healthy lifestyle score stratified by preexisting macrovascular disease. CI, confidence interval; HR, hazard ratio.
(DOCX)

S15 Table HRs (95% CIs) of microvascular complications according to the healthy lifestyle score among individuals with T2D using 2 competing risk models. CI, confidence interval; HR, hazard ratio; T2D, type 2 diabetes.
(DOCX)

## Abbreviations

ALP: alkaline phosphatase
ALT: alanine aminotransferase
AST: aspartate aminotransferase
BMI: body mass index
CI: confidence interval
CRIC: Chronic Renal Insufficiency Cohort
CRP: C-reactive protein
CVD: cardiovascular disease
DBP: diastolic blood pressure
DIY: do-it-yourself
eGFR: estimated glomerular filtration rate
GGT: gamma glutamyltransferase
HDL-C: high-density lipoprotein cholesterol
HR: hazard ratio
LDL-C: low-density lipoprotein cholesterol
MET: metabolic equivalent of task
ONTARGET: Ongoing Telmisartan Alone and in Combination with Ramipril Global Endpoint Trial
PAF: population-attributable fraction
SBP: systolic blood pressure
TC: total cholesterol
T2D: type 2 diabetes
WC: waist circumference
