# Supplementary figures and images for: Healthy lifestyle behaviors, mediating biomarkers, and risk of microvascular complications among individuals with type 2 diabetes: A cohort study
Source: PLoS Med. 2023 Jan 10;20(1):e1004135. doi: 10.1371/journal.pmed.1004135 (PMC9831321; doi:10.1371/journal.pmed.1004135)

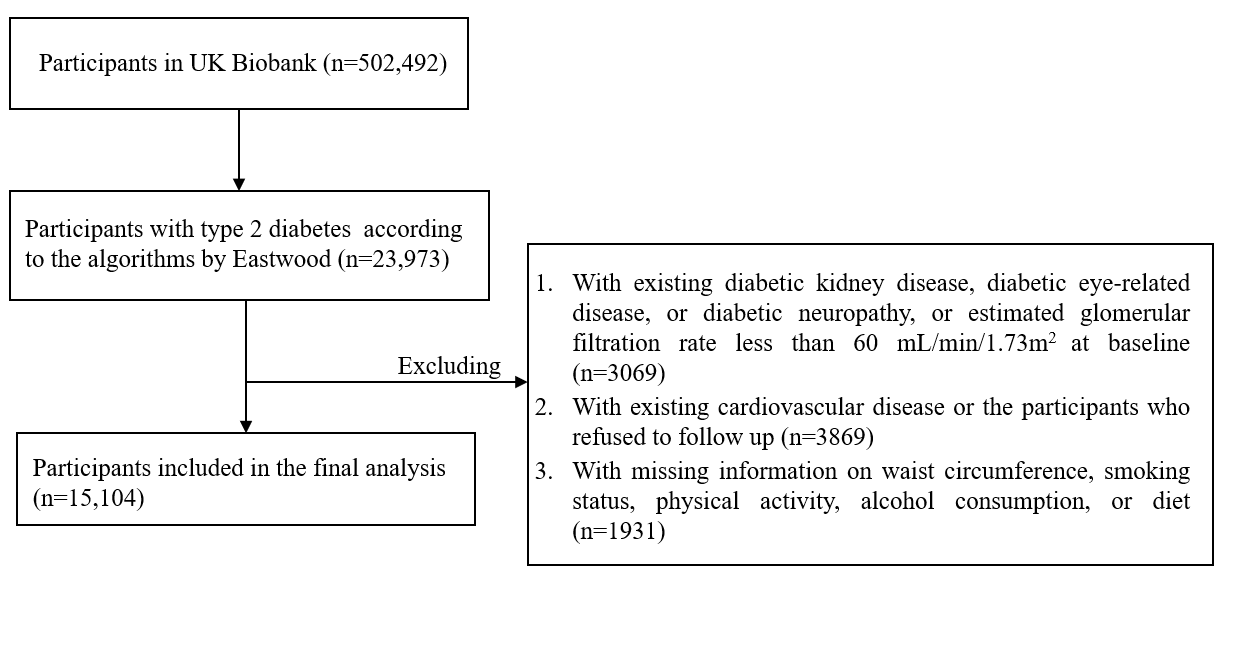

Supplement: S1 Fig — (TIF) [file pmed.1004135.s002.tif]

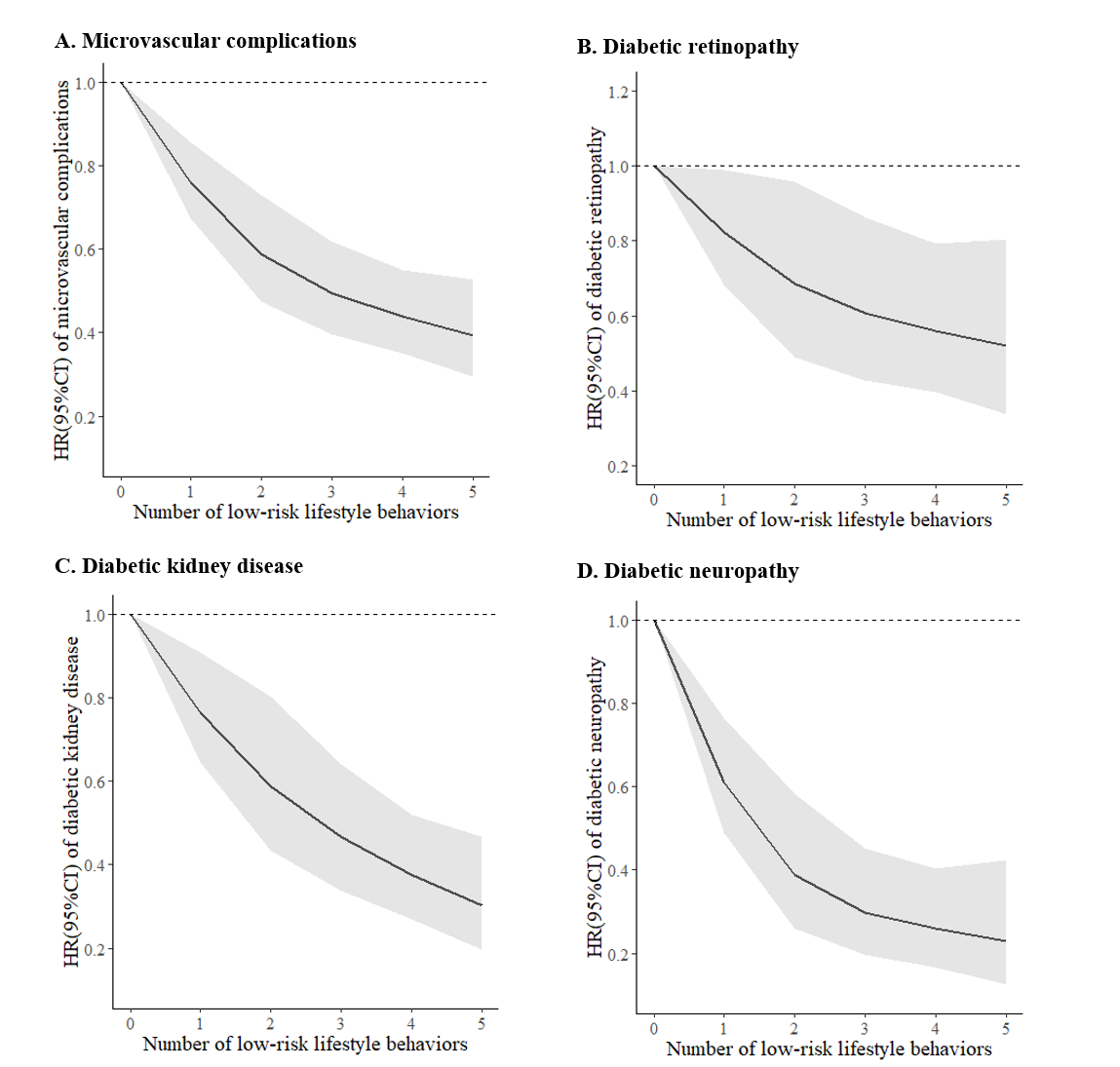

Supplement: S2 Fig — X-axis showed the numbers of low-risk lifestyle behaviors, and y-axis showed the HRs of the composite microvascular complications (A), diabetic retinopathy (B), diabetic kidney disease (C), and diabetic neuropathy (D). Black curves were HRs, and grey zones were 95% CIs. All P-nonlinearity were ≥0.10, and all P for overall association were <0.001 (except for diabetic retinopathy: P for overall association = 0.005). CI, confidence interval; HR, hazard ratio; T2D, type 2 diabetes. (TIF) [file pmed.1004135.s003.tif]

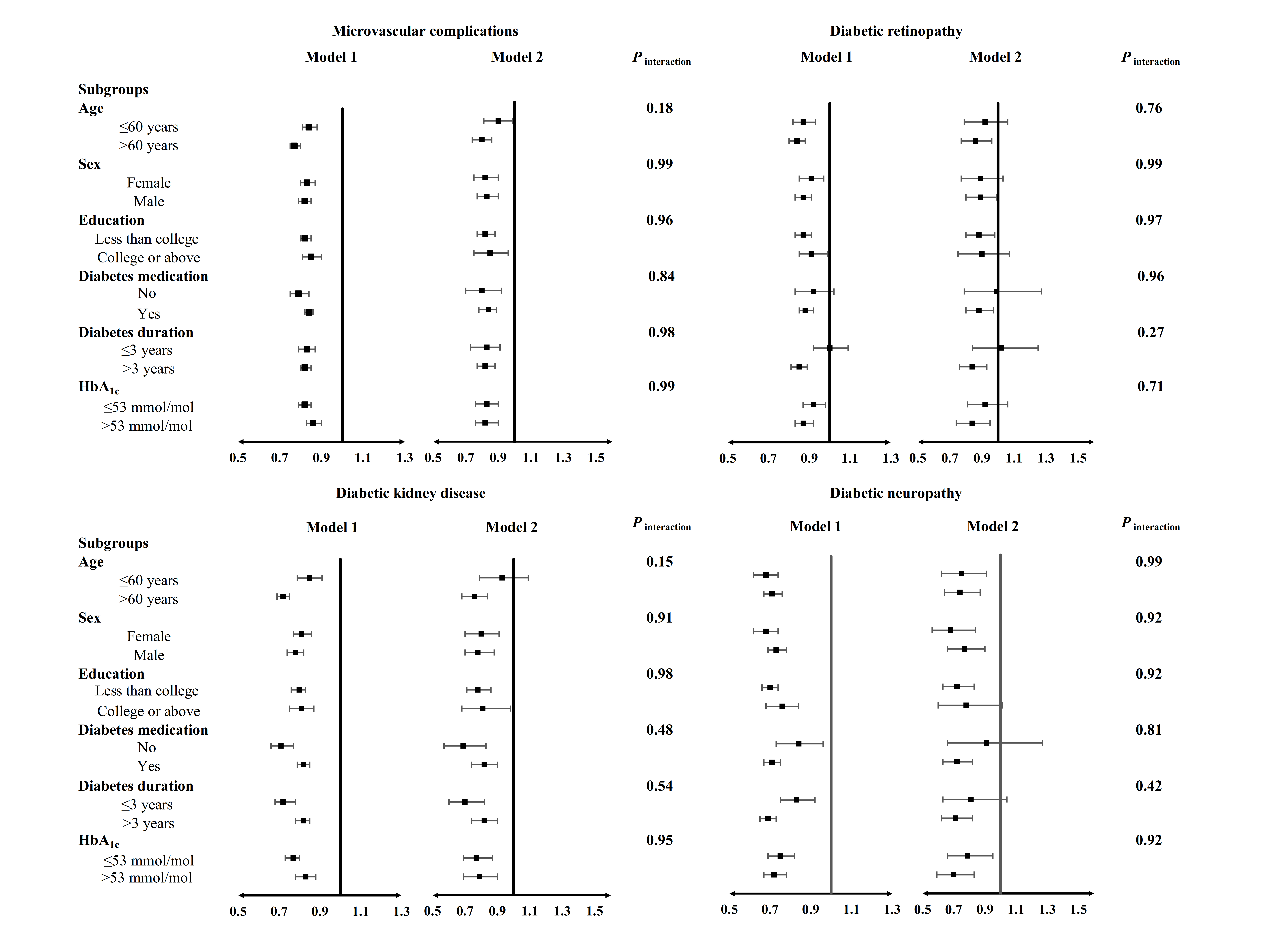

Supplement: S3 Fig — Model 1: unadjusted model. Model 2: age (continuous, years), sex (male, female), race (White, others), education attainment (college or university degree, A/AS levels or equivalent or O levels/GCSEs or equivalent or other professional qualifications, or none of the above), Townsend Deprivation Index (continuous), sleep duration (<6, 6–8, or ≥9 hours/day), family history of CVD (yes, no), family history of hypertension (yes, no), prevalence of hypertension (yes, no), diabetes duration (continuous, years), use of diabetes medication (none, only oral medication pills, or insulin or others), HbA1c (continuous, mmol/mol), use of antihypertensive medication (yes, no), use of lipid-lowing medication (yes, no), and use of aspirin (yes, no), with exception of stratifying factors. Interactions were tested based on Model 2. (TIF) [file pmed.1004135.s004.tif]
