# Supplementary material for: Healthy lifestyle behaviors, mediating biomarkers, and risk of microvascular complications among individuals with type 2 diabetes: A cohort study
Source: PLoS Med. 2023 Jan 10;20(1):e1004135. doi: 10.1371/journal.pmed.1004135 (PMC9831321; doi:10.1371/journal.pmed.1004135)
Supplement: S1 Table — (DOCX) [file pmed.1004135.s005.docx]

**S1 Table.** Percentage of missing values of the covariates

| **Covariates** | **Missing percentage** |
| --- | --- |
| Race/Ethnicity | 0.5% |
| Townsend deprivation index | 0.2% |
| Education | 1.4% |
| HbA_1c_, mmol/mol | 7.0% |
| Prevalence of hypertension | 0.1% |
| Diabetes duration, years | 1.6% |
| Sleep duration, hours/day | 0.8% |
