# Supplementary material for: Healthy lifestyle behaviors, mediating biomarkers, and risk of microvascular complications among individuals with type 2 diabetes: A cohort study
Source: PLoS Med. 2023 Jan 10;20(1):e1004135. doi: 10.1371/journal.pmed.1004135 (PMC9831321; doi:10.1371/journal.pmed.1004135)
Supplement: S2 Table — (DOCX) [file pmed.1004135.s006.docx]

**S2 Table.** Baseline characteristics between individuals with type 2 diabetes included in the study and those excluded due to the missing values

| **Characteristics** | **Included** | **Excluded** | ***P*** |
| --- | --- | --- | --- |
| Number of patients | 15,104 | 1931 |  |
| Age, years | 59.3 ± 7.1 | 59.9 ± 7.1 | <0.001 |
| Men | 9112 (60.3) | 1026 (53.1) | <0.001 |
| Ethnicity, White | 13,207 (87.4) | 1484 (76.9) | <0.001 |
| Townsend deprivation index | -0.6 ± 3.4 | 0.1 ± 3.6 | <0.001 |
| College or university degree | 3925 (26.0) | 290 (15.0) | <0.001 |
| Current smokers | 1593 (10.5) | 372 (19.3) | <0.001 |
| Healthy waist circumference (<94 cm for men, or <80 cm for women) | 2164 (14.3) | 250 (12.9) | 0.10 |
| Physically active (top third of total physical activity) | 5043 (33.4) | 318 (16.5) | <0.001 |
| Moderate alcohol intake (1-28 g/day for men, or 1-14 g/day for women) | 9875 (65.4) | 998 (51.7) | <0.001 |
| Healthy diet (≥5 dietary components at ideal levels) | 3282 (21.7) | 277 (14.3) | <0.001 |
| HbA_1c_, mmol/mol | 51.9 ± 13.3 | 51.9 ± 14.0 | 0.68 |
| Family history of CVD | 8484 (56.2) | 993 (51.4) | <0.001 |
| Family history of hypertension | 6179 (40.9) | 675 (35.0) | <0.001 |
| Prevalence of hypertension | 10,041 (66.5) | 1289 (66.8) | 0.33 |
| Diabetes duration, years | 6.4 ± 9.0 | 9.0 ± 13.7 | <0.001 |
| Sleep duration (7-8 hours/day) | 9071 (60.1) | 1043 (54.0) | 0.36 |
| Use of diabetes medication | 10,318 (68.3) | 1335 (69.1) | 0.50 |
| Use of antihypertensive medication | 8909 (59.0) | 1033 (53.5) | <0.001 |
| Use of lipid-lowing medication | 10,857 (71.9) | 1206 (62.5) | <0.001 |
| Use of aspirin | 6679 (44.2) | 829 (42.9) | 0.28 |

Data are presented as mean (SD) for continuous variable and n (%) for categorical variables.
