# Supplementary material for: Healthy lifestyle behaviors, mediating biomarkers, and risk of microvascular complications among individuals with type 2 diabetes: A cohort study
Source: PLoS Med. 2023 Jan 10;20(1):e1004135. doi: 10.1371/journal.pmed.1004135 (PMC9831321; doi:10.1371/journal.pmed.1004135)
Supplement: S3 Table — CI, confidence interval; HR, hazard ratio; T2D, type 2 diabetes. (DOCX) [file pmed.1004135.s007.docx]

**S3 Table.** HRs (95% CIs) of microvascular complications according to individual lifestyle behaviors in individuals with type 2 diabetes

|  | **Microvascular complications** | | | **Diabetic retinopathy** | | | **Diabetic kidney disease** | | | **Diabetic neuropathy** | | |
| --- | --- | --- | --- | --- | --- | --- | --- | --- | --- | --- | --- | --- |
|  | Person  -years | Cases | HR (95% CI) | Person  -years | Cases | HR (95% CI) | Person  -years | Cases | HR (95% CI) | Person  -years | Cases | HR (95% CI) |
| **Model 1** |  |  |  |  |  |  |  |  |  |  |  |  |
| ***Waist circumference*** |  |  |  |  |  |  |  |  |  |  |  |  |
| ≥94cm (men) or  ≥80cm (women) | 105,571 | 1156 | 1.00 | 102,359 | 486 | 1.00 | 102,648 | 576 | 1.00 | 103,001 | 282 | 1.00 |
| < 94cm (men) or  < 80cm (women) | 16,874 | 140 | 0.76 (0.70, 0.82) | 17,055 | 72 | 0.93 (0.83, 1.04) | 17,210 | 49 | 0.53 (0.46, 0.60) | 17,237 | 33 | 0.76 (0.65, 0.90) |
| ***Smoking status*** |  |  |  |  |  |  |  |  |  |  |  |  |
| Current smoker | 12,185 | 144 | 1.00 | 12,402 | 57 | 1.00 | 12,419 | 77 | 1.00 | 12,479 | 37 | 1.00 |
| Non-current smoker | 105,260 | 1152 | 0.94 (0.87, 1.02) | 107,012 | 501 | 1.04 (0.92, 1.18) | 107,439 | 548 | 0.82 (0.74, 0.92) | 107,759 | 278 | 0.91 (0.78, 1.06) |
| ***Physical activity*** |  |  |  |  |  |  |  |  |  |  |  |  |
| Bottom two thirds | 77,880 | 937 | 1.00 | 79,347 | 399 | 1.00 | 79,603 | 457 | 1.00 | 79,842 | 243 | 1.00 |
| Top third | 39,565 | 359 | 0.78 (0.74, 0.83) | 40,067 | 159 | 0.81 (0.74, 0.88) | 40,254 | 168 | 0.77 (0.71, 0.84) | 40,396 | 72 | 0.62 (0.55, 0.70) |
| ***Dietary score*** |  |  | 1 |  |  |  |  |  |  |  |  |  |
| < 5 ideal components | 91,750 | 1026 | 1.00 | 93,251 | 438 | 1.00 | 93,591 | 497 | 1.00 | 93,903 | 249 | 1.00 |
| ≥5 ideal components | 25,695 | 270 | 0.96 (0.91, 1.02) | 26,163 | 120 | 0.99 (0.91, 1.09) | 26,266 | 128 | 0.96 (0.88, 1.04) | 26,335 | 66 | 0.99 (0.88, 1.12) |
| ***Alcohol intake*** |  |  |  |  |  |  |  |  |  |  |  |  |
| Others | 39,646 | 526 | 1.00 | 40,501 | 223 | 1.00 | 40,666 | 246 | 1.00 | 40,741 | 151 | 1.00 |
| 1-28 g/day (men) or  1-14 g/day (women) | 77,799 | 770 | 0.74 (0.71, 0.78) | 78,913 | 335 | 0.77 (0.71, 0.83) | 79,192 | 379 | 0.79 (0.74, 0.85) | 79,497 | 164 | 0.57 (0.51, 0.62) |
| **Model 2** |  |  |  |  |  |  |  |  |  |  |  |  |
| ***Waist circumference*** |  |  |  |  |  |  |  |  |  |  |  |  |
| ≥94cm (men) or  ≥80cm (women) | 105,571 | 1156 | 1.00 | 102,359 | 486 | 1.00 | 102,648 | 576 | 1.00 | 103,001 | 282 | 1.00 |
| < 94cm (men) or  < 80cm (women) | 16,874 | 140 | 0.79 (0.66, 0.95) | 17,055 | 72 | 0.88 (0.68, 1.13) | 17,210 | 49 | 0.59 (0.44, 0.80) | 17,237 | 33 | 0.86 (0.59, 1.25) |
| ***Smoking status*** |  |  |  |  |  |  |  |  |  |  |  |  |
| Current smoker | 12,185 | 144 | 1.00 | 12,402 | 57 | 1.00 | 12,419 | 77 | 1.00 | 12,479 | 37 | 1.00 |
| Non-current smoker | 105,260 | 1152 | 0.93 (0.78, 1.11) | 107,012 | 501 | 1.06 (0.81, 1.41) | 107,439 | 548 | 0.76 (0.60, 0.97) | 107,759 | 278 | 0.95 (0.67, 1.36) |
| ***Physical activity*** |  |  |  |  |  |  |  |  |  |  |  |  |
| Bottom two thirds | 77,880 | 937 | 1.00 | 79,347 | 399 | 1.00 | 79,603 | 457 | 1.00 | 79,842 | 243 | 1.00 |
| Top third | 39,565 | 359 | 0.81 (0.72, 0.92) | 40,067 | 159 | 0.86 (0.71, 1.04) | 40,254 | 168 | 0.78 (0.65, 0.93) | 40,396 | 72 | 0.66 (0.51, 0.87) |
| ***Dietary score*** |  |  | 1 |  |  |  |  |  |  |  |  |  |
| < 5 ideal components | 91,750 | 1026 | 1.00 | 93,251 | 438 | 1.00 | 93,591 | 497 | 1.00 | 93,903 | 249 | 1.00 |
| ≥5 ideal components | 25,695 | 270 | 0.92 (0.80, 1.05) | 26,163 | 120 | 0.94 (0.77, 1.16) | 26,266 | 128 | 0.91 (0.74, 1.10) | 26,335 | 66 | 0.99 (0.75, 1.30) |
| ***Alcohol intake*** |  |  |  |  |  |  |  |  |  |  |  |  |
| Others | 39,646 | 526 | 1.00 | 40,501 | 223 | 1.00 | 40,666 | 246 | 1.00 | 40,741 | 151 | 1.00 |
| 1-28 g/day (men) or  1-14 g/day (women) | 77,799 | 770 | 0.73 (0.65, 0.82) | 78,913 | 335 | 0.77 (0.65, 0.91) | 79,192 | 379 | 0.79 (0.67, 0.92) | 79,497 | 164 | 0.54 (0.43, 0.68) |

**Model 1:** individual lifestyle factors were mutually adjusted

**Model 2:** age (continuous, years), sex (male, female), ethnicity (White, others), education attainment (college or university degree, A/AS levels or equivalent or O levels/GCSEs or equivalent or other professional qualifications, or none of the above), Townsend Deprivation Index (continuous), sleep duration (<6, 6-8, or ≥9 hours/day), family history of CVD (yes, no), family history of hypertension (yes, no), prevalence of hypertension (yes, no), diabetes duration (continuous, years), use of diabetes medication (none, only oral medication pills, or insulin or others), use of antihypertensive medication (yes, no), use of lipid-lowing medication (yes, no), use of aspirin (yes, no), and HbA_1c_ (continuous, mmol/mol). Individual lifestyle factors were mutually adjusted.
