# Supplementary material for: Healthy lifestyle behaviors, mediating biomarkers, and risk of microvascular complications among individuals with type 2 diabetes: A cohort study
Source: PLoS Med. 2023 Jan 10;20(1):e1004135. doi: 10.1371/journal.pmed.1004135 (PMC9831321; doi:10.1371/journal.pmed.1004135)
Supplement: S4 Table — (DOCX) [file pmed.1004135.s008.docx]

**S4 Table.** Multivariable-adjusted linear regression models for the association between the healthy lifestyle score and biomarker levels among individuals with type 2 diabetes

|  | **Healthy lifestyle score** | | | | | | | | |
| --- | --- | --- | --- | --- | --- | --- | --- | --- | --- |
|  | **Model 1** | | | |  | **Model 2** | | | |
| **Biomarkers** | **β** | **Lower 95%CI** | **Upper 95%CI** | ***P*- value** |  | **β** | **Lower 95%CI** | **Upper 95%CI** | ***P*- value** |
| **Biomarkers of renal function** |  |  |  |  |  |  |  |  |  |
| Cystatin C (mg/L) | -0.1278 | -0.1440 | -0.1116 | <0.001 |  | -0.1549 | -0.1715 | -0.1383 | <0.001 |
| Creatinine (μmol/L) | 0.0924 | 0.0762 | 0.1087 | <0.001 |  | 0.0249 | 0.0057 | 0.0441 | 0.01 |
| Urate (μmol/L) | -0.0437 | -0.0600 | -0.0273 | <0.001 |  | -0.0788 | -0.0957 | -0.0618 | <0.001 |
| Urea (mmol/L) | 0.1107 | 0.0945 | 0.1270 | <0.001 |  | 0.0769 | 0.0604 | 0.0934 | <0.001 |
| **Biomarkers of liver function** |  |  |  |  |  |  |  |  |  |
| Alanine aminotransferase (U/L) | -0.0476 | -0.0639 | -0.0313 | <0.001 |  | -0.0552 | -0.0718 | -0.0386 | <0.001 |
| Alkaline phosphatase (U/L) | -0.1021 | -0.1184 | -0.0859 | <0.001 |  | -0.0653 | -0.0816 | -0.0490 | <0.001 |
| Aspartate aminotransferase (U/L) | -0.0235 | -0.0399 | -0.0071 | 0.005 |  | -0.0359 | -0.0519 | -0.0198 | <0.001 |
| Gamma glutamyltransferase (U/L) | -0.1646 | -0.1807 | -0.1485 | <0.001 |  | -0.1644 | -0.1807 | -0.1481 | <0.001 |
| Total bilirubin (μmol/L) | 0.1121 | 0.0958 | 0.1284 | <0.001 |  | 0.0627 | 0.0460 | 0.0794 | <0.001 |
| Total protein (g/L) | -0.0364 | -0.0535 | -0.0193 | <0.001 |  | -0.0157 | -0.0326 | 0.0012 | 0.07 |
| Albumin (g/L) | 0.0860 | 0.0690 | 0.1030 | <0.001 |  | 0.0675 | 0.0506 | 0.0844 | <0.001 |
| **Biomarkers of lipid profile** |  |  |  |  |  |  |  |  |  |
| Total blood cholesterol (mmol/L) | -0.0435 | -0.0599 | -0.0272 | <0.001 |  | -0.0448 | -0.0622 | -0.0274 | <0.001 |
| HDL-cholesterol (mmol/L) | 0.0923 | 0.0752 | 0.1093 | <0.001 |  | 0.0998 | 0.0822 | 0.1174 | <0.001 |
| LDL-cholesterol (mmol/L) | -0.0554 | -0.0717 | -0.0390 | <0.001 |  | -0.0604 | -0.0778 | -0.0429 | <0.001 |
| Triglycerides (mmol/L) | -0.1392 | -0.1554 | -0.1230 | <0.001 |  | -0.1327 | -0.1489 | -0.1166 | <0.001 |
| Apolipoprotein A (g/L) | 0.0654 | 0.0484 | 0.0825 | <0.001 |  | 0.0723 | 0.0547 | 0.0900 | <0.001 |
| Apolipoprotein B (g/L) | -0.0846 | -0.1010 | -0.0682 | <0.001 |  | -0.0843 | -0.1013 | -0.0672 | <0.001 |
| Lipoprotein A (nmol/L) | -0.0010 | -0.0198 | 0.0177 | 0.92 |  | 0.0127 | -0.0055 | 0.0308 | 0.17 |
| **Inflammatory biomarkers** |  |  |  |  |  |  |  |  |  |
| C-reactive protein (mg/L) | -0.2250 | -0.2409 | -0.2090 | <0.001 |  | -0.1824 | -0.1988 | -0.1660 | <0.001 |
| White blood cell count (x10^^9^/L) | -0.1722 | -0.1882 | -0.1563 | <0.001 |  | -0.1416 | -0.1573 | -0.1259 | <0.001 |
| **Blood pressure indices** |  |  |  |  |  |  |  |  |  |
| Systolic blood pressure (mmHg) | 0.0198 | 0.0034 | 0.0361 | 0.02 |  | -0.0014 | -0.0178 | 0.0151 | 0.87 |
| Diastolic blood pressure (mmHg) | -0.0575 | -0.0738 | -0.0412 | <0.001 |  | -0.0489 | -0.0651 | -0.0328 | <0.001 |
| **Biomarkers of glucose metabolism** | | | | | | | | | |
| HbA_1c_ (mmol/mol) | -0.0702 | -0.0860 | -0.0545 | <0.001 |  | -0.0589 | -0.0753 | -0.0425 | <0.001 |

**Model 1**: unadjusted model.

**Model 2**: age (continuous, years), sex (male, female), ethnicity (White, others), education attainment (college or university degree, A/AS levels or equivalent or O levels/GCSEs or equivalent or other professional qualifications, or none of the above), Townsend Deprivation Index (continuous), sleep duration (<6, 6-8, or ≥9 hours/day), family history of CVD (yes, no), family history of hypertension (yes, no), prevalence of hypertension (yes, no), diabetes duration (continuous, years), use of diabetes medication (none, only oral medication pills, or insulin or others), HbA_1c_ (continuous, mmol/mol), use of antihypertensive medication, use of lipid-lowing medication, and use of aspirin (yes, no).

For biomarkers of systolic blood pressure and diastolic blood pressure, model 2 was not adjusted for prevalence of hypertension (yes, no). HbA_1c_ (continuous, mmol/mol) levels were not adjusted when HbA_1c_ was analyzed as a biomarker in model 2.

The levels of biomarkers were nature log transformed except for systolic blood pressure and diastolic blood pressure.
