# Supplementary material for: Healthy lifestyle behaviors, mediating biomarkers, and risk of microvascular complications among individuals with type 2 diabetes: A cohort study
Source: PLoS Med. 2023 Jan 10;20(1):e1004135. doi: 10.1371/journal.pmed.1004135 (PMC9831321; doi:10.1371/journal.pmed.1004135)
Supplement: S5 Table — (DOCX) [file pmed.1004135.s009.docx]

**S5 Table.** Risk estimates of microvascular complications associated with the selected biomarkers (1-SD increment) among individuals with type 2 diabetes

|  | **Microvascular complications** | | | | **Diabetic retinopathy** | | | | **Diabetic kidney disease** | | | | **Diabetic neuropathy** | | | |
| --- | --- | --- | --- | --- | --- | --- | --- | --- | --- | --- | --- | --- | --- | --- | --- | --- |
|  | HR | Lower | Upper | ***P*** | HR | Lower | Upper | ***P*** | HR | Lower | Upper | ***P*** | HR | Lower | Upper | ***P*** |
| **Model 1** |  |  |  |  |  |  |  |  |  |  |  |  |  |  |  |  |
| Cystatin C (mg/L) ^*^ | - | - | - | - | 1.15 | 1.05 | 1.25 | 0.002 | - | - | - | - | 1.40 | 1.26 | 1.57 | <0.001 |
| Creatinine (μmol/L) ^*^ | - | - | - | - | 1.02 | 0.93 | 1.11 | 0.67 | - | - | - | - | 0.99 | 0.88 | 1.11 | 0.88 |
| Urate (μmol/L) ^*^ | - | - | - | - | 0.88 | 0.81 | 0.96 | 0.004 | - | - | - | - | 1.03 | 0.91 | 1.15 | 0.67 |
| Urea (mmol/L) ^*^ | - | - | - | - | 1.11 | 1.02 | 1.22 | 0.02 | - | - | - | - | 1.12 | 1.00 | 1.26 | 0.05 |
| Alanine aminotransferase (U/L) ^*^ | 0.90 | 0.85 | 0.96 | <0.001 | 0.87 | 0.80 | 0.95 | 0.002 | 0.85 | 0.78 | 0.92 | <0.001 | 1.01 | 0.90 | 1.13 | 0.90 |
| Alkaline phosphatase (U/L) ^*^ | 1.07 | 1.01 | 1.13 | 0.02 | 1.03 | 0.94 | 1.12 | 0.52 | 1.07 | 0.98 | 1.16 | 0.13 | 1.11 | 0.99 | 1.24 | 0.09 |
| Aspartate aminotransferase (U/L) ^*^ | 1.00 | 0.94 | 1.05 | 0.91 | 0.90 | 0.82 | 0.99 | 0.02 | 0.97 | 0.90 | 1.06 | 0.52 | 1.11 | 1.00 | 1.24 | 0.05 |
| Gamma glutamyltransferase (U/L) ^*^ | 1.05 | 0.99 | 1.11 | 0.12 | 0.93 | 0.85 | 1.02 | 0.10 | 1.06 | 0.97 | 1.15 | 0.20 | 1.22 | 1.10 | 1.36 | <0.001 |
| Total bilirubin (μmol/L) ^*^ | 0.93 | 0.87 | 0.98 | 0.01 | 0.99 | 0.91 | 1.08 | 0.80 | 0.92 | 0.85 | 1.01 | 0.07 | 0.86 | 0.76 | 0.97 | 0.01 |
| Albumin (g/L) ^*^ | 0.77 | 0.73 | 0.82 | <0.001 | 0.81 | 0.74 | 0.89 | <0.001 | 0.70 | 0.65 | 0.76 | <0.001 | 0.75 | 0.67 | 0.84 | <0.001 |
| Total blood cholesterol (mmol/L) ^*^ | 0.86 | 0.82 | 0.91 | <0.001 | 0.86 | 0.79 | 0.94 | <0.001 | 0.84 | 0.78 | 0.91 | <0.001 | 0.82 | 0.73 | 0.92 | <0.001 |
| HDL-cholesterol (mmol/L) ^*^ | 0.90 | 0.85 | 0.96 | <0.001 | 0.98 | 0.90 | 1.08 | 0.75 | 0.86 | 0.79 | 0.94 | <0.001 | 0.80 | 0.70 | 0.90 | <0.001 |
| LDL-cholesterol (mmol/L) ^*^ | 0.86 | 0.81 | 0.91 | <0.001 | 0.84 | 0.77 | 0.92 | <0.001 | 0.84 | 0.78 | 0.92 | <0.001 | 0.84 | 0.75 | 0.94 | 0.002 |
| Triglycerides (mmol/L) ^*^ | 1.07 | 1.01 | 1.14 | 0.02 | 0.94 | 0.86 | 1.02 | 0.15 | 1.11 | 1.02 | 1.21 | 0.01 | 1.26 | 1.12 | 1.41 | <0.001 |
| Apolipoprotein A (g/L) ^*^ | 0.93 | 0.88 | 0.99 | 0.02 | 0.96 | 0.87 | 1.05 | 0.35 | 0.92 | 0.85 | 1.00 | 0.06 | 0.83 | 0.74 | 0.94 | 0.003 |
| Apolipoprotein B (g/L) ^*^ | 0.87 | 0.82 | 0.92 | <0.001 | 0.85 | 0.78 | 0.93 | <0.001 | 0.87 | 0.80 | 0.94 | <0.001 | 0.85 | 0.76 | 0.96 | 0.01 |
| C-reactive protein (mg/L) ^*^ | 1.10 | 1.04 | 1.17 | 0.001 | 0.97 | 0.88 | 1.06 | 0.47 | 1.20 | 1.10 | 1.30 | <0.001 | 1.24 | 1.10 | 1.39 | <0.001 |
| White blood cell count (x10^^9^/L) ^*^ | 1.04 | 0.99 | 1.11 | 0.14 | 1.00 | 0.91 | 1.09 | 0.92 | 1.14 | 1.05 | 1.23 | 0.002 | 0.90 | 0.80 | 1.01 | 0.07 |
| Diastolic blood pressure (mmHg) | 0.91 | 0.86 | 0.96 | <0.001 | 0.88 | 0.80 | 0.96 | 0.003 | 0.92 | 0.85 | 1.00 | 0.06 | 0.91 | 0.81 | 1.03 | 0.13 |
| HbA_1c_ (mmol/mol) | 1.38 | 1.31 | 1.45 | <0.001 | 1.60 | 1.48 | 1.72 | <0.001 | 1.22 | 1.13 | 1.31 | <0.001 | 1.55 | 1.41 | 1.72 | <0.001 |
| **Model 2** |  |  |  |  |  |  |  |  |  |  |  |  |  |  |  |  |
| Cystatin C (mg/L) ^*^ | - | - | - | - | 1.09 | 1.00 | 1.19 | 0.048 | - | - | - | - | 1.26 | 1.13 | 1.41 | <0.001 |
| Creatinine (μmol/L) ^*^ | - | - | - | - | 1.01 | 0.91 | 1.12 | 0.84 | - | - | - | - | 0.92 | 0.81 | 1.06 | 0.26 |
| Urate (μmol/L) ^*^ | - | - | - | - | 1.02 | 0.93 | 1.11 | 0.70 | - | - | - | - | 1.13 | 1.00 | 1.27 | 0.06 |
| Urea (mmol/L) ^*^ | - | - | - | - | 1.05 | 0.96 | 1.15 | 0.28 | - | - | - | - | 1.01 | 0.90 | 1.14 | 0.85 |
| Alanine aminotransferase (U/L) ^*^ | 0.91 | 0.86 | 0.97 | 0.002 | 0.88 | 0.80 | 0.96 | 0.01 | 0.75 | 0.63 | 0.9 | 0.002 | 0.99 | 0.88 | 1.11 | 0.85 |
| Alkaline phosphatase (U/L) ^*^ | 1.01 | 0.95 | 1.07 | 0.68 | 0.95 | 0.87 | 1.04 | 0.24 | 1.15 | 0.86 | 1.54 | 0.34 | 1.02 | 0.91 | 1.15 | 0.74 |
| Aspartate aminotransferase (U/L) ^*^ | 1.00 | 0.95 | 1.06 | 0.87 | 0.92 | 0.84 | 1.01 | 0.07 | 0.96 | 0.76 | 1.21 | 0.73 | 1.10 | 0.99 | 1.22 | 0.07 |
| Gamma glutamyltransferase (U/L) ^*^ | 1.05 | 0.99 | 1.11 | 0.11 | 0.94 | 0.86 | 1.03 | 0.18 | 1.09 | 0.97 | 1.23 | 0.17 | 1.18 | 1.06 | 1.31 | 0.003 |
| Total bilirubin (μmol/L) ^*^ | 0.95 | 0.89 | 1.01 | 0.11 | 1.04 | 0.95 | 1.14 | 0.37 | 0.87 | 0.7 | 1.08 | 0.19 | 0.86 | 0.76 | 0.98 | 0.02 |
| Albumin (g/L) ^*^ | 0.82 | 0.77 | 0.87 | <0.001 | 0.90 | 0.82 | 0.99 | 0.03 | 0.01 | 0.001 | 0.02 | <0.001 | 0.81 | 0.72 | 0.92 | <0.001 |
| Total blood cholesterol (mmol/L) ^*^ | 0.94 | 0.89 | 1.00 | 0.07 | 0.94 | 0.86 | 1.04 | 0.22 | 0.70 | 0.47 | 1.05 | 0.08 | 0.89 | 0.78 | 1.00 | 0.06 |
| HDL-cholesterol (mmol/L) ^*^ | 0.90 | 0.84 | 0.95 | <0.001 | 1.00 | 0.91 | 1.10 | 0.98 | 0.47 | 0.33 | 0.68 | <0.001 | 0.83 | 0.73 | 0.95 | 0.01 |
| LDL-cholesterol (mmol/L) ^*^ | 0.95 | 0.90 | 1.01 | 0.13 | 0.93 | 0.84 | 1.02 | 0.12 | 0.83 | 0.60 | 1.14 | 0.24 | 0.91 | 0.80 | 1.03 | 0.13 |
| Triglycerides (mmol/L) ^*^ | 1.07 | 1.01 | 1.14 | 0.02 | 0.96 | 0.88 | 1.05 | 0.40 | 1.24 | 1.06 | 1.46 | 0.01 | 1.21 | 1.07 | 1.35 | 0.002 |
| Apolipoprotein A (g/L) ^*^ | 0.92 | 0.87 | 0.98 | 0.01 | 0.97 | 0.88 | 1.07 | 0.55 | 0.47 | 0.28 | 0.80 | 0.01 | 0.85 | 0.75 | 0.97 | 0.02 |
| Apolipoprotein B (g/L) ^*^ | 0.96 | 0.90 | 1.02 | 0.16 | 0.92 | 0.84 | 1.01 | 0.09 | 0.89 | 0.63 | 1.27 | 0.53 | 0.91 | 0.80 | 1.03 | 0.13 |
| C-reactive protein (mg/L) ^*^ | 1.11 | 1.05 | 1.18 | <0.001 | 0.96 | 0.87 | 1.05 | 0.35 | 1.21 | 1.12 | 1.31 | <0.001 | 1.22 | 1.08 | 1.38 | 0.002 |
| White blood cell count (x10^^9^/L) ^*^ | 1.00 | 0.94 | 1.06 | 0.96 | 0.96 | 0.88 | 1.05 | 0.36 | 1.34 | 0.98 | 1.83 | 0.06 | 0.85 | 0.77 | 0.95 | 0.004 |
| Diastolic blood pressure (mmHg) | 0.97 | 0.92 | 1.03 | 0.37 | 0.94 | 0.86 | 1.03 | 0.19 | 1.00 | 0.99 | 1.01 | 0.97 | 0.97 | 0.86 | 1.09 | 0.56 |
| HbA_1c_ (mmol/mol) | 1.27 | 1.20 | 1.35 | <0.001 | 1.39 | 1.27 | 1.51 | <0.001 | 1.69 | 1.19 | 2.40 | 0.003 | 1.37 | 1.22 | 1.54 | <0.001 |

**Model 1**: healthy lifestyle scores (continuous).

**Model 2**: age (continuous, years), sex (male, female), ethnicity (White, others), education attainment (college or university degree, A/AS levels or equivalent or O levels/GCSEs or equivalent or other professional qualifications, or none of the above), Townsend Deprivation Index (continuous), sleep duration (<6, 6-8, or ≥9 hours/day), family history of CVD (yes, no), family history of hypertension (yes, no), prevalence of hypertension (yes, no), diabetes duration (continuous, years), use of diabetes medication (none, only oral medication pills, or insulin or others), HbA_1c_ (continuous, mmol/mol), use of antihypertensive medication, use of lipid-lowing medication, use of aspirin (yes, no), and healthy lifestyle scores (continuous).

For biomarker of diastolic blood pressure, model 2 was not adjusted for prevalence of hypertension (yes, no). HbA_1c_ (continuous, mmol/mol) levels were not adjusted when HbA_1c_ was analyzed as a biomarker in model 2.

The levels of biomarkers were nature log-transformed before analyses except for diastolic blood pressure.

The subsequent mediation analysis was based on the model 2.
