# Supplementary material for: Healthy lifestyle behaviors, mediating biomarkers, and risk of microvascular complications among individuals with type 2 diabetes: A cohort study
Source: PLoS Med. 2023 Jan 10;20(1):e1004135. doi: 10.1371/journal.pmed.1004135 (PMC9831321; doi:10.1371/journal.pmed.1004135)
Supplement: S6 Table — CI, confidence interval; HR, hazard ratio; T2D, type 2 diabetes. (DOCX) [file pmed.1004135.s010.docx]

**S6 Table.** HRs (95% CIs) of microvascular complications according to different combinations of low-risk lifestyle factors among individuals with type 2 diabetes

|  | **Microvascular complications** | | |  | **Diabetic retinopathy** | | |  | **Diabetic kidney disease** | | |  | **Diabetic neuropathy** | | |
| --- | --- | --- | --- | --- | --- | --- | --- | --- | --- | --- | --- | --- | --- | --- | --- |
|  | Person-years | Cases | HR (95% CI) |  | Person-years Person-year | Cases | HR (95% CI) |  | Person-years Person-year | Cases | HR (95% CI) |  | Person-years Person-year | Cases | HR (95% CI) |
| **Model 1** | | | | | | | | | | | | | | | |
| Two low-risk factors (physical activity, diet) | | | | | | | | | | | | | | | |
| 0 | 62,410 | 759 | 1.00 |  | 63,533 | 325 | 1.00 |  | 63,786 | 369 | 1.00 |  | 63,959 | 194 | 1.00 |
| 1-2 | 55,035 | 537 | 0.83 (0.79, 0.87) |  | 55,881 | 233 | 0.83 (0.77, 0.89) |  | 56,072 | 256 | 0.83 (0.77, 0.89) |  | 56,279 | 121 | 0.74 (0.67, 0.82) |
| Three low-risk factors (above two plus smoking) | | | | | | | | | | | | | | | |
| 0 | 7387 | 101 | 1.00 |  | 7531 | 41 | 1.00 |  | 7558 | 53 | 1.00 |  | 7584 | 28 | 1.00 |
| 1 | 59,216 | 694 | 0.85 (0.77, 0.93) |  | 60,259 | 299 | 0.91 (0.79, 1.06) |  | 60,484 | 334 | 0.76 (0.67, 0.87) |  | 60,656 | 174 | 0.78 (0.65, 0.93) |
| 2-3 | 50,842 | 501 | 0.73 (0.67, 0.81) |  | 51,624 | 218 | 0.79 (0.68, 0.92) |  | 51,816 | 238 | 0.66 (0.58, 0.76) |  | 51,998 | 113 | 0.62 (0.51, 0.74) |
| Four low-risk factors (above three plus alcohol consumption) | | | | | | | | | | | | | | | |
| 0 | 2863 | 49 | 1.00 |  | 2932 | 22 | 1.00 |  | 2940 | 25 | 1.00 |  | 2954 | 15 | 1.00 |
| 1 | 25,672 | 355 | 0.78 (0.69, 0.90) |  | 26,241 | 140 | 0.70 (0.58, 0.86) |  | 26,320 | 173 | 0.73 (0.60, 0.88) |  | 26,368 | 103 | 0.75 (0.59, 0.96) |
| 2 | 50,732 | 538 | 0.59 (0.52, 0.68) |  | 51,546 | 239 | 0.61 (0.50, 0.74) |  | 51,763 | 256 | 0.54 (0.45, 0.65) |  | 51,910 | 126 | 0.46 (0.36, 0.59) |
| 3-4 | 38,178 | 354 | 0.53 (0.46, 0.60) |  | 38,695 | 157 | 0.53 (0.44, 0.65) |  | 38,834 | 171 | 0.50 (0.41, 0.60) |  | 39,005 | 71 | 0.35 (0.28, 0.45) |
| Five low-risk factors (above four plus waist circumference) | | | | | | | | | | | | | | | |
| 0 | 2312 | 41 | 1.00 |  | 2374  4 | 17 | 1.00 |  | 2377 | 22 | 1.00 |  | 2385 | 14 | 1.00 |
| 1 | 23,372 | 337 | 0.80 (0.70, 0.93) |  | 23,909 | 132 | 0.77 (0.61, 0.96) |  | 23,982 | 166 | 0.68 (0.45, 1.04) |  | 24,024 | 96 | 0.68 (0.53, 0.87) |
| 2 | 47,476 | 507 | 0.59 (0.51, 0.68) |  | 48,250 | 224 | 0.64 (0.51, 0.80) |  | 48,401 | 245 | 0.50 (0.33, 0.76) |  | 48,553 | 122 | 0.42 (0.33, 0.54) |
| 3 | 31,985 | 312 | 0.53 (0.46, 0.62) |  | 32,442 | 139 | 0.59 (0.47, 0.74) |  | 32,554 | 153 | 0.49 (0.40, 0.59) |  | 32,742 | 61 | 0.31 (0.24, 0.41) |
| 4 -5 | 12,301 | 99 | 0.44 (0.37, 0.52) |  | 12,439 | 46 | 0.51 (0.40, 0.65) |  | 12,542 | 39 | 0.32 (0.25, 0.41) |  | 12,533 | 22 | 0.29 (0.22, 0.40) |
| **Model 2** | | | | | | | | | | | | | | | |
| Two low-risk factors (physical activity, diet) | | | | | | | | | | | | | | | |
| 0 | 62,410 | 759 | 1.00 |  | 63,533 | 325 | 1.00 |  | 63,786 | 369 | 1.00 |  | 63,959 | 194 | 1.00 |
| 1-2 | 55,035 | 537 | 0.83 (0.74, 0.93) |  | 55,881 | 233 | 0.84 (0.71, 1.00) |  | 56,072 | 256 | 0.81 (0.69, 0.96) |  | 56,279 | 121 | 0.78 (0.62, 0.99) |
| Three low-risk factors (above two plus smoking) | | | | | | | |  |  |  |  |  |  |  |  |
| 0 | 7387 | 101 | 1.00 |  | 8204 | 44 | 1.00 |  | 7558 | 53 | 1.00 |  | 7584 | 28 | 1.00 |
| 1 | 59,216 | 694 | 0.84 (0.68, 1.03) |  | 62,154 | 309 | 0.92 (0.66, 1.28) |  | 60,484 | 334 | 0.71 (0.53, 0.95) |  | 60,656 | 174 | 0.83 (0.55, 1.24) |
| 2-3 | 50,842 | 501 | 0.73 (0.58, 0.90) |  | 54,096 | 222 | 0.81 (0.58, 1.14) |  | 51,816 | 238 | 0.60 (0.44, 0.81) |  | 51,998 | 113 | 0.69 (0.45, 1.05) |
| Four low-risk factors (above three plus alcohol consumption) | | | | | | | | | | | | | | | |
| 0 | 2863 | 49 | 1.00 |  | 2932 | 22 | 1.00 |  | 2940 | 25 | 1.00 |  | 2954 | 15 | 1.00 |
| 1 | 25,672 | 355 | 0.78 (0.58, 1.05) |  | 26,241 | 140 | 0.70 (0.45, 1.10) |  | 26,320 | 173 | 0.68 (0.45, 1.04) |  | 26,368 | 103 | 0.79 (0.45, 1.36) |
| 2 | 50,732 | 538 | 0.59 (0.44, 0.79) |  | 51,546 | 239 | 0.61 (0.39, 0.95) |  | 51,763 | 256 | 0.50 (0.33, 0.76) |  | 51,910 | 126 | 0.49 (0.28, 0.84) |
| 3-4 | 38,178 | 354 | 0.52 (0.38, 0.70) |  | 38,695 | 157 | 0.55 (0.35, 0.86) |  | 38,834 | 171 | 0.45 (0.29, 0.69) |  | 39,005 | 71 | 0.38 (0.21, 0.67) |
| Five low-risk factors (above four plus waist circumference) | | | | | | | | | | | | | | | |
| 0 | 2312 | 41 | 1.00 |  | 2374  4 | 17 | 1.00 |  | 2377 | 22 | 1.00 |  | 2385 | 14 | 1.00 |
| 1 | 23,372 | 337 | 0.81 (0.58, 1.12) |  | 23,909 | 132 | 0.77 (0.46, 1.28) |  | 23,982 | 166 | 0.70 (0.44, 1.09) |  | 24,024 | 96 | 0.71 (0.40, 1.25) |
| 2 | 47,476 | 507 | 0.59 (0.43, 0.81) |  | 48,250 | 224 | 0.65 (0.39, 1.06) |  | 48,401 | 245 | 0.50 (0.32, 0.77) |  | 48,553 | 122 | 0.45 (0.25, 0.78) |
| 3 | 31,985 | 312 | 0.54 (0.38, 0.74) |  | 32,442 | 139 | 0.60 (0.36, 1.00) |  | 32,554 | 153 | 0.46 (0.29, 0.72) |  | 32,742 | 61 | 0.34 (0.19, 0.62) |
| 4 -5 | 12,301 | 99 | 0.45 (0.31, 0.65) |  | 12,439 | 46 | 0.51 (0.29, 0.90) |  | 12,542 | 39 | 0.31 (0.18, 0.52) |  | 12,533 | 22 | 0.34 (0.17, 0.67) |

**Model 1**: only the individual behaviors mutually adjusted.

**Model 2**: age (continuous, years), sex (male, female), ethnicity (White, others), education attainment (college or university degree, A/AS levels or equivalent or O levels/GCSEs or equivalent or other professional qualifications, or none of the above), Townsend Deprivation Index (continuous), sleep duration (<6, 6-8, or ≥9 hours/day), family history of CVD (yes, no), family history of hypertension (yes, no), prevalence of hypertension (yes, no), diabetes duration (continuous, years), use of diabetes medication (none, only oral medication pills, or insulin or others), HbA_1c_ (continuous, mmol/mol), use of antihypertensive medication (yes, no), use of lipid-lowing medication (yes, no), and use of aspirin (yes, no) with the individual behaviors mutually adjusted.
