# Supplementary material for: Healthy lifestyle behaviors, mediating biomarkers, and risk of microvascular complications among individuals with type 2 diabetes: A cohort study
Source: PLoS Med. 2023 Jan 10;20(1):e1004135. doi: 10.1371/journal.pmed.1004135 (PMC9831321; doi:10.1371/journal.pmed.1004135)
Supplement: S8 Table — CI, confidence interval; HR, hazard ratio; T2D, type 2 diabetes. (DOCX) [file pmed.1004135.s012.docx]

**S8 Table.** HRs (95% CIs) of microvascular complications according to the healthy lifestyle score using moderate drinking and non-drinking as the low-risk behavior in individuals with type 2 diabetes

|  | **Number of low-risk lifestyle factors** | | | | | **HR _continuous_** |
| --- | --- | --- | --- | --- | --- | --- |
|  | **0-1** | **2** | **3** | **4-5** | ***P_-_*_trend_** |  |
| **Microvascular complications** | | | | | | |
| Cases/person-years | 196/14,089 | 596/51,577 | 376/36,970 | 128/14,809 |  |  |
| Model 1 | 1 | 0.83 (0.77, 0.89) | 0.73 (0.67, 0.79) | 0.62 (0.56, 0.68) | <0.001 | 0.86 (0.84, 0.89) |
| Model 2 | 1 | 0.80 (0.68, 0.94) | 0.70 (0.59, 0.84) | 0.61 (0.49, 0.77) | <0.001 | 0.86 (0.81, 0.91) |
| **Diabetic retinopathy** |  |  |  |  |  |  |
| Cases/person-years | 78/14,380 | 259/52,485 | 158/37,564 | 63/14,984 |  |  |
| Model 1 | 1 | 0.91 (0.81, 1.02) | 0.78 (0.69, 0.88) | 0.78 (0.67, 0.90) | <0.001 | 0.90 (0.87, 0.94) |
| Model 2 | 1 | 0.86 (0.66, 1.11) | 0.73 (0.55, 0.96) | 0.73 (0.52, 1.03) | 0.02 | 0.89 (0.81, 0.98) |
| **Diabetic kidney disease** |  |  |  |  |  |  |
| Cases/person-years | 100/14,420 | 285/52,678 | 190/37,632 | 50/15,128 |  |  |
| Model 1 | 1 | 0.78 (0.70, 0.86) | 0.73 (0.65, 0.81) | 0.47 (0.41, 0.55) | <0.001 | 0.82 (0.79, 0.85) |
| Model 2 | 1 | 0.75 (0.59, 0.94) | 0.70 (0.55, 0.90) | 0.47 (0.34, 0.67) | <0.001 | 0.82 (0.75, 0.90) |
| **Diabetic neuropathy** |  |  |  |  |  |  |
| Cases/person-years | 56/14,461 | 147/52,811 | 86/37,832 | 26/15,134 |  |  |
| Model 1 | 1 | 0.72 (0.63, 0.83) | 0.59 (0.51, 0.68) | 0.44 (0.36, 0.55) | <0.001 | 0.78 (0.74, 0.83) |
| Model 2 | 1 | 0.74 (0.54, 1.01) | 0.63 (0.45, 0.89) | 0.51 (0.32, 0.82) | 0.002 | 0.82 (0.72, 0.93) |

**Model 1**: unadjusted model.

**Model 2**: age (continuous, years), sex (male, female), ethnicity (White, others), education attainment (college or university degree, A/AS levels or equivalent or O levels/GCSEs or equivalent or other professional qualifications, or none of the above), Townsend Deprivation Index (continuous), sleep duration (<6, 6-8, or ≥9 hours/day), family history of CVD (yes, no), family history of hypertension (yes, no), prevalence of hypertension (yes, no), diabetes duration (continuous, years), use of diabetes medication (none, only oral medication pills, or insulin or others), HbA_1c_ (continuous, mmol/mol), use of antihypertensive medication (yes, no), use of lipid-lowing medication (yes, no), and use of aspirin (yes, no).

Low-risk alcohol drinking was defined as <28 g/d for men and <14 g/d of ethanol intakes for women.
