# Supplementary material for: Healthy lifestyle behaviors, mediating biomarkers, and risk of microvascular complications among individuals with type 2 diabetes: A cohort study
Source: PLoS Med. 2023 Jan 10;20(1):e1004135. doi: 10.1371/journal.pmed.1004135 (PMC9831321; doi:10.1371/journal.pmed.1004135)
Supplement: S9 Table — BMI, body mass index; CI, confidence interval; HR, hazard ratio; T2D, type 2 diabetes; WHR, waist-to-hip ratio. (DOCX) [file pmed.1004135.s013.docx]

**S9 Table.** HRs (95% CIs) of microvascular complications according to the healthy lifestyle score using optimal body mass index (BMI) or optimal waist-to-hip ratio (WHR) as the low-risk behavior in individuals with type 2 diabetes

|  | **Number of low-risk lifestyle factors** | | | | | **HR _continuous_** |
| --- | --- | --- | --- | --- | --- | --- |
|  | **0-1** | **2** | **3** | **4-5** | ***P_-_*_trend_** |  |
| **Using optimal BMI instead of waist circumstance as the low-risk behavior** | | | | | | |
| ***Microvascular complications*** | | | | | | |
| Cases/person-years | 377/25,837 | 525/48,791 | 300/31,862 | 87/10,525 |  |  |
| Model 1 | 1 | 0.73 (0.68, 0.77) | 0.63 (0.59, 0.68) | 0.56 (0.50, 0.62) | <0.001 | 0.82 (0.80, 0.84) |
| Model 2 | 1 | 0.72 (0.63, 0.82) | 0.62 (0.53, 0.73) | 0.55 (0.43, 0.69) | <0.001 | 0.81 (0.76, 0.86) |
| ***Diabetic retinopathy*** |  |  |  |  |  |  |
| Cases/person-years | 153/26,425 | 230/49,593 | 135/32,300 | 39/10,647 |  |  |
| Model 1 | 1 | 0.79 (0.72, 0.87) | 0.71 (0.64, 0.79) | 0.62 (0.53, 0.73) | <0.001 | 0.86 (0.82, 0.89) |
| Model 2 | 1 | 0.79 (0.65, 0.98) | 0.72 (0.56, 0.91) | 0.61 (0.42, 0.87) | 0.001 | 0.85 (0.78, 0.93) |
| ***Diabetic kidney disease*** |  |  |  |  |  |  |
| Cases/person-years | 183/26,520 | 250/49,775 | 151/32,389 | 36/10,734 |  |  |
| Model 1 | 1 | 0.71 (0.65, 0.78) | 0.66 (0.60, 0.73) | 0.47 (0.40, 0.55) | <0.001 | 0.80 (0.77, 0.84) |
| Model 2 | 1 | 0.71 (0.58, 0.86) | 0.64 (0.51, 0.80) | 0.46 (0.32, 0.67) | <0.001 | 0.79 (0.73, 0.87) |
| ***Diabetic neuropathy*** |  |  |  |  |  |  |
| Cases/person-years | 110/26,575 | 125/49,915 | 56/32,580 | 21/10,726 |  |  |
| Model 1 | 1 | 0.60 (0.53, 0.67) | 0.41 (0.36, 0.47) | 0.47 (0.38, 0.58) | <0.001 | 0.71 (0.68, 0.75) |
| Model 2 | 1 | 0.61 (0.47, 0.79) | 0.43 (0.31, 0.60) | 0.51 (0.31, 0.82) | <0.001 | 0.73 (0.64, 0.83) |
| **Using optimal WHR instead of waist circumstance as the low-risk behavior** | | | | | | |
| ***Microvascular complications*** | | | | | | |
| Cases/person-years | 366/24,429 | 500/46,170 | 327/33,684 | 102/13,114 |  |  |
| Model 1 | 1 | 0.71 (0.67, 0.76) | 0.63 (0.59, 0.68) | 0.51 (0.46, 0.56) | <0.001 | 0.81 (0.79, 0.83) |
| Model 2 | 1 | 0.71 (0.62, 0.81) | 0.65 (0.56, 0.75) | 0.53 (0.42, 0.66) | <0.001 | 0.82 (0.78, 0.87) |
| ***Diabetic retinopathy*** |  |  |  |  |  |  |
| Cases/person-years | 142/25,026 | 221/46,922 | 152/34,150 | 43/13,265 |  |  |
| Model 1 | 1 | 0.82 (0.75, 0.90) | 0.77 (0.70, 0.86) | 0.56 (0.48, 0.66) | <0.001 | 0.86 (0.83, 0.89) |
| Model 2 | 1 | 0.84 (0.68, 1.04) | 0.81 (0.64, 1.03) | 0.60 (0.42, 0.85) | 0.01 | 0.88 (0.80, 0.96) |
| ***Diabetic kidney disease*** |  |  |  |  |  |  |
| Cases/person-years | 183/25,064 | 238/47,152 | 158/34,252 | 45/13,340 |  |  |
| Model 1 | 1 | 0.68 (0.62, 0.74) | 0.61 (0.56, 0.68) | 0.45 (0.39, 0.52) | <0.001 | 0.79 (0.76, 0.82) |
| Model 2 | 1 | 0.67 (0.55, 0.81) | 0.62 (0.50, 0.77) | 0.46 (0.33, 0.65) | <0.001 | 0.79 (0.73, 0.86) |
| ***Diabetic neuropathy*** |  |  |  |  |  |  |
| Cases/person-years | 109/25,112 | 121/47,281 | 64/34,454 | 21/13,339 |  |  |
| Model 1 | 1 | 0.58 (0.52, 0.66) | 0.42 (0.37, 0.48) | 0.36 (0.29, 0.44) | <0.001 | 0.70 (0.66, 0.73) |
| Model 2 | 1 | 0.59 (0.45, 0.77) | 0.45 (0.33, 0.62) | 0.41 (0.25, 0.66) | <0.001 | 0.72 (0.64, 0.82) |

**Model 1**: unadjusted model.

**Model 2**: age (continuous, years), sex (male, female), ethnicity (White, others), education attainment (college or university degree, A/AS levels or equivalent or O levels/GCSEs or equivalent or other professional qualifications, or none of the above), Townsend Deprivation Index (continuous), sleep duration (<6, 6-8, or ≥9 hours/day), family history of CVD (yes, no), family history of hypertension (yes, no), prevalence of hypertension (yes, no), diabetes duration (continuous, years), use of diabetes medication (none, only oral medication pills, or insulin or others), HbA_1c_ (continuous, mmol/mol), use of antihypertensive medication (yes, no), use of lipid-lowing medication (yes, no), and use of aspirin (yes, no).

Optimal BMI was defined as 18.5-24.9 kg/m^2^ in 15,046 individuals with type 2 diabetes who have BMI data.

Optimal WHR was defined as <0.90 for men and <0.85 for women in 15,098 individuals with type 2 diabetes who have WHR data.
