# Supplementary material for: Healthy lifestyle behaviors, mediating biomarkers, and risk of microvascular complications among individuals with type 2 diabetes: A cohort study
Source: PLoS Med. 2023 Jan 10;20(1):e1004135. doi: 10.1371/journal.pmed.1004135 (PMC9831321; doi:10.1371/journal.pmed.1004135)
Supplement: S10 Table — CI, confidence interval; HR, hazard ratio; T2D, type 2 diabetes. (DOCX) [file pmed.1004135.s014.docx]

**S10 Table.** HRs (95% CIs) of microvascular complications according to the weighted lifestyle score in individuals with type 2 diabetes

|  | **Weighted lifestyle score** | | | | | |
| --- | --- | --- | --- | --- | --- | --- |
|  | **Group 1 (0-1.2)** | **Group 2 (1.2-2.2)** | **Group 3 (2.2-3.4)** | **Group 4 (3.4-5.0)** | ***P*_-trend_** | **HR _continuous_** |
| **Microvascular complications** | |  |  |  |  |  |
| Cases/person-years | 378/24,840 | 508/48,251 | 311/32,137 | 99/12,218 | - | - |
| Unadjusted | 1 | 0.68 (0.64, 0.72) | 0.62 (0.58, 0.66) | 0.52 (0.47, 0.57) | <0.001 | 0.83 (0.81, 0.85) |
| Model 1 | 1 | 0.68 (0.60, 0.78) | 0.61 (0.52, 0.71) | 0.50 (0.40, 0.63) | <0.001 | 0.82 (0.78, 0.86) |
| Model 2 | 1 | 0.69 (0.60, 0.79) | 0.62 (0.53, 0.73) | 0.53 (0.42, 0.66) | <0.001 | 0.83 (0.79, 0.87) |
| Model 3 | 1 | 0.70 (0.61, 0.80) | 0.63 (0.54, 0.74) | 0.54 (0.43, 0.67) | <0.001 | 0.83 (0.79, 0.88) |
| **Diabetic retinopathy** |  |  |  |  |  |  |
| Cases/person-years | 153/25,489 | 224/48,965 | 136/32,597 | 45/12,363 | - | - |
| Unadjusted | 1 | 0.75 (0.69, 0.82) | 0.68 (0.62, 0.76) | 0.60 (0.51, 0.69) | <0.001 | 0.87 (0.84, 0.90) |
| Model 1 | 1 | 0.77 (0.63, 0.95) | 0.69 (0.54, 0.87) | 0.59 (0.42, 0.83) | <0.001 | 0.87 (0.81, 0.94) |
| Model 2 | 1 | 0.78 (0.63, 0.96) | 0.70 (0.55, 0.88) | 0.60 (0.43, 0.85) | <0.001 | 0.87 (0.81, 0.94) |
| Model 3 | 1 | 0.79 (0.64, 0.98) | 0.70 (0.55, 0.89) | 0.61 (0.44, 0.86) | <0.001 | 0.87 (0.81, 0.94) |
| **Diabetic kidney disease** |  |  |  |  |  |  |
| Cases/person-years | 180/25,553 | 253/49,159 | 153/32,686 | 39/12,460 | - | - |
| Unadjusted | 1 | 0.71 (0.65, 0.78) | 0.64 (0.58, 0.71) | 0.43 (0.37, 0.50) | <0.001 | 0.81 (0.79, 0.84) |
| Model 1 | 1 | 0.71 (0.59, 0.87) | 0.61 (0.49, 0.76) | 0.40 (0.28, 0.57) | <0.001 | 0.79 (0.74, 0.85) |
| Model 2 | 1 | 0.73 (0.60, 0.89) | 0.64 (0.52, 0.80) | 0.43 (0.31, 0.62) | <0.001 | 0.81 (0.76, 0.87) |
| Model 3 | 1 | 0.74 (0.61, 0.90) | 0.66 (0.53, 0.83) | 0.45 (0.32, 0.64) | <0.001 | 0.82 (0.76, 0.88) |
| **Diabetic neuropathy** |  |  |  |  |  |  |
| Cases/person-years | 118/25,582 | 112/49,319 | 62/32,892 | 23/12,444 | - | - |
| Unadjusted | 1 | 0.49 (0.43, 0.55) | 0.40 (0.35, 0.46) | 0.39 (0.32, 0.48) | <0.001 | 0.72 (0.69, 0.76) |
| Model 1 | 1 | 0.47 (0.36, 0.61) | 0.39 (0.28, 0.53) | 0.38 (0.24, 0.60) | <0.001 | 0.72 (0.65, 0.79) |
| Model 2 | 1 | 0.49 (0.38, 0.64) | 0.42 (0.30, 0.57) | 0.44 (0.28, 0.70) | <0.001 | 0.74 (0.67, 0.82) |
| Model 3 | 1 | 0.49 (0.38, 0.64) | 0.41 (0.30, 0.57) | 0.42 (0.27, 0.67) | <0.001 | 0.73 (0.66, 0.81) |

**Model 1:** age (continuous, years), sex (male or female), Townsend Deprivation Index (continuous), and race/ethnicity (White, others).

**Model 2:** **Model 1** + education attainment (college or university degree, A/AS levels or equivalent or O levels/GCSEs or equivalent or other professional qualifications, or none of the above), sleep duration (<6, 6-8, or ≥9 hours/day), family history of CVD (yes, no), family history of hypertension (yes, no), and prevalence of hypertension (yes, no).

**Model 3: Model 2** + diabetes duration (continuous, years), HbA_1c_ (continuous, mmol/mol), use of diabetes medication (none, only oral medication pills, or insulin or others), use of antihypertensive medication (yes, no), use of lipid-lowing medication (yes, no), and use of aspirin (yes, no).

The weighted lifestyle score= 5 x [ (β_1_ x behavior1 + β_2_ x behavior2 + β_3_ x behavior3 + β_4_ x behavior4 + β_5_ x behavior5) / (sum of the β coefficients of 5 healthy lifestyle behaviors)].

β coefficients of each lifestyle behaviors with the composite microvascular complications were based on the Cox proportional hazards regression model that included all the covariates in Model 3 with all five lifestyle factors (coded as binary variables) mutually adjusted.

β_1_ = -0.23 for low risk of waist circumference (<94 cm for men, or <80 cm for women), β_2_ = -0.21 for low risk of physical activity (top third of total physical activity), β_3_= -0.07 for low risk of smoking (non-current smoking), β_4_= -0.08 for low risk of diet (≥5 dietary components at ideal levels), and β_5_ = -0.31 for low risk of alcohol intake (1-28 g/day for men; 1-14 g/day for women).
