# Supplementary material for: Healthy lifestyle behaviors, mediating biomarkers, and risk of microvascular complications among individuals with type 2 diabetes: A cohort study
Source: PLoS Med. 2023 Jan 10;20(1):e1004135. doi: 10.1371/journal.pmed.1004135 (PMC9831321; doi:10.1371/journal.pmed.1004135)
Supplement: S11 Table — CI, confidence interval; eGFR, estimated glomerular filtration rate; HR, hazard ratio; T2D, type 2 diabetes. (DOCX) [file pmed.1004135.s015.docx]

**S11 Table.** HRs (95% CIs) of diabetic kidney disease according to the healthy lifestyle score among individuals with type 2 diabetes with additional adjustment for eGFR

| **Diabetic kidney disease** | **Number of low-risk lifestyle factors** | | | | | **Continuous** |
| --- | --- | --- | --- | --- | --- | --- |
|  | **0-1** | **2** | **3** | **4-5** | ***P*_-trend_** |  |
|  |  |  |  |  |  |  |
| HRs (95% CIs) | 1 | 0.65 (0.54, 0.79) | 0.61 (0.49, 0.76) | 0.46 (0.32, 0.65) | <0.001 | 0.78 (0.71, 0.85) |

HRs were adjusted for age (continuous, years), sex (male, female), ethnicity (White, others), education attainment (college or university degree, A/AS levels or equivalent or O levels/GCSEs or equivalent or other professional qualifications, or none of the above), Townsend Deprivation Index (continuous), sleep duration (<6, 6-8, or ≥9 hours/day), family history of CVD (yes, no), family history of hypertension (yes, no), prevalence of hypertension (yes, no), diabetes duration (continuous, years), use of diabetes medication (none, only oral medication pills, or insulin or others), HbA_1c_ (continuous, mmol/mol), use of antihypertensive medication (yes, no), use of lipid-lowing medication (yes, no), use of aspirin (yes, no), and eGFR (continuous, mL/min/1.73m^2^).
