# Supplementary material for: Healthy lifestyle behaviors, mediating biomarkers, and risk of microvascular complications among individuals with type 2 diabetes: A cohort study
Source: PLoS Med. 2023 Jan 10;20(1):e1004135. doi: 10.1371/journal.pmed.1004135 (PMC9831321; doi:10.1371/journal.pmed.1004135)
Supplement: S12 Table — eGFR, estimated glomerular filtration rate; T2D, type 2 diabetes. (DOCX) [file pmed.1004135.s016.docx]

**S12 Table.** Possible mediators of the associations between the healthy lifestyle score and diabetic kidney disease among individuals with type 2 diabetes with additional adjustment for eGFR^*^

|  | **Total effect** | | | | **Natural direct effect** | | | | **Natural indirect effect** | | | | **Proportion mediated** | |
| --- | --- | --- | --- | --- | --- | --- | --- | --- | --- | --- | --- | --- | --- | --- |
|  | **Beta** | **Lower** | **Upper** | ***P*** | **Beta** | **Lower** | **Upper** | ***P*** | **Beta** | **Lower** | **Upper** | ***P*** | **% (95%CI)** ^†^ | ***P*** |
| Albumin (g/L) | -0.0089 | -0.0159 | -0.0035 | 0.002 | -0.0079 | -0.0148 | -0.0027 | 0.002 | -0.0010 | -0.0014 | -0.0006 | <0.001 | 10.69 (5.71, 23.34) | 0.002 |
| HDL-C (mmol/L) | -0.0096 | -0.0166 | -0.0038 | <0.001 | -0.0089 | -0.0158 | -0.0031 | <0.001 | -0.0007 | -0.0012 | -0.0002 | 0.01 | 7.08 (2.10, 17.67) | 0.01 |
| Triglycerides (mmol/L) | -0.0116 | -0.0185 | -0.0056 | <0.001 | -0.0110 | -0.0179 | -0.0050 | <0.001 | -0.0007 | -0.0013 | -0.0003 | 0.06 | 5.75 (-0.24, 15.28) | 0.06 |
| Apolipoprotein A (mg/dL) | -0.0094 | -0.0162 | -0.0039 | <0.001 | -0.0091 | -0.0158 | -0.0037 | <0.001 | -0.0003 | -0.0007 | 0.00003 | 0.08 | 3.28 (-0.34, 9.37) | 0.08 |
| C-reactive protein (mg/L) | -0.0119 | -0.0190 | -0.0061 | <0.001 | -0.0102 | -0.0168 | -0.0044 | 0.002 | -0.0018 | -0.0027 | -0.0009 | <0.001 | 14.77 (7.16, 27.65) | <0.001 |
| HbA_1c_ (mmol/mol) | -0.0127 | -0.0194 | -0.0067 | <0.001 | -0.0120 | -0.0186 | -0.0060 | <0.001 | -0.0007 | -0.0011 | -0.0004 | <0.001 | 5.77 (2.66, 11.50) | <0.001 |

Estimates were adjusted for age (continuous, years), sex (male, female), ethnicity (White, others), education attainment (college or university degree, A/AS levels or equivalent or O levels/GCSEs or equivalent or other professional qualifications, or none of the above), Townsend Deprivation Index (continuous), sleep duration (<6, 6-8, or ≥9 hours/day), family history of CVD (yes, no), family history of hypertension (yes, no), prevalence of hypertension (yes, no), diabetes duration (continuous, years), use of diabetes medication (none, only oral medication pills, or insulin or others), HbA_1c_ (continuous, mmol/mol), use of antihypertensive medication (yes, no), use of lipid-lowing medication (yes, no), use of aspirin (yes, no), and eGFR (mL/min/1.73m^2^).

HbA_1c_ (continuous, mmol/mol) levels were not adjusted when HbA_1c_ was analyzed as a mediator.

^*^The levels of biomarkers were nature log-transformed before analyses.

^†^ 1000 Bootstrap resampling
