# Supplementary material for: Healthy lifestyle behaviors, mediating biomarkers, and risk of microvascular complications among individuals with type 2 diabetes: A cohort study
Source: PLoS Med. 2023 Jan 10;20(1):e1004135. doi: 10.1371/journal.pmed.1004135 (PMC9831321; doi:10.1371/journal.pmed.1004135)
Supplement: S13 Table — CI, confidence interval; HR, hazard ratio; T2D, type 2 diabetes. (DOCX) [file pmed.1004135.s017.docx]

**S13 Table.** HRs (95% CIs) of microvascular complications according to the healthy lifestyle score in 18,501 individuals with type 2 diabetes including patients with pre-existing macrovascular disease

|  | **Number of low-risk lifestyle factors** | | | | | **HR _continuous_** |
| --- | --- | --- | --- | --- | --- | --- |
|  | **0-1** | **2** | **3** | **4-5** | ***P_-_*_trend_** |  |
| **Microvascular complications** | | | | | | |
| Cases/person-years | 546/31,835 | 726/57,516 | 439/38,293 | 131/14,671 |  |  |
| Model 1 | 1 | 0.72 (0.69, 0.76) | 0.65 (0.62, 0.69) | 0.51 (0.47, 0.55) | <0.001 | 0.82 (0.80, 0.83) |
| Model 2 | 1 | 0.73 (0.65, 0.82) | 0.68 (0.60, 0.77) | 0.55 (0.45, 0.67) | <0.001 | 0.83 (0.79, 0.87) |
| **Diabetic retinopathy** |  |  |  |  |  |  |
| Cases/person-years | 198/32,736 | 293/58,669 | 176/38,909 | 63/14,848 |  |  |
| Model 1 | 1 | 0.82 (0.75, 0.89) | 0.74 (0.68, 0.81) | 0.69 (0.61, 0.79) | <0.001 | 0.89 (0.86, 0.92) |
| Model 2 | 1 | 0.84 (0.70, 1.00) | 0.77 (0.62, 0.95) | 0.72 (0.54, 0.97) | 0.01 | 0.90 (0.83, 0.97) |
| **Diabetic kidney disease** |  |  |  |  |  |  |
| Cases/person-years | 290/32,783 | 383/58,775 | 236/38,992 | 56/14,978 |  |  |
| Model 1 | 1 | 0.72 (0.67, 0.77) | 0.67 (0.62, 0.72) | 0.41 (0.36, 0.47) | <0.001 | 0.79 (0.74, 0.84) |
| Model 2 | 1 | 0.75 (0.62, 0.85) | 0.69 (0.58, 0.82) | 0.45 (0.33, 0.60) | <0.001 | 0.80 (0.75, 0.86) |
| **Diabetic neuropathy** |  |  |  |  |  |  |
| Cases/person-years | 158/32,864 | 172/59,090 | 81/39,277 | 29/14,998 |  |  |
| Model 1 | 1 | 0.60 (0.54, 0.66) | 0.42 (0.38, 0.48) | 0.40 (0.33, 0.48) | <0.001 | 0.70 (0.67, 0.73) |
| Model 2 | 1 | 0.62 (0.50, 0.77) | 0.47 (0.36, 0.62) | 0.47 (0.31, 0.70) | <0.001 | 0.73 (0.66, 0.81) |

**Model 1**: unadjusted model.

**Model 2**: age (continuous, years), sex (male, female), ethnicity (White, others), education attainment (college or university degree, A/AS levels or equivalent or O levels/GCSEs or equivalent or other professional qualifications, or none of the above), Townsend Deprivation Index (continuous), sleep duration (<6, 6-8, or ≥9 hours/day), family history of CVD (yes, no), family history of hypertension (yes, no), prevalence of hypertension (yes, no), diabetes duration (continuous, years), use of diabetes medication (none, only oral medication pills, or insulin or others), HbA_1c_ (continuous, mmol/mol), use of antihypertensive medication (yes, no), use of lipid-lowing medication (yes, no), use of aspirin (yes, no), and prevalence of macrovascular disease (yes, no).
