# Supplementary material for: Healthy lifestyle behaviors, mediating biomarkers, and risk of microvascular complications among individuals with type 2 diabetes: A cohort study
Source: PLoS Med. 2023 Jan 10;20(1):e1004135. doi: 10.1371/journal.pmed.1004135 (PMC9831321; doi:10.1371/journal.pmed.1004135)
Supplement: S14 Table — CI, confidence interval; HR, hazard ratio. (DOCX) [file pmed.1004135.s018.docx]

**S14 Table.** HRs (95% CIs) of microvascular complications according to the healthy lifestyle score stratified by pre-existing macrovascular disease

|  | **Number of low-risk lifestyle factors** | | | | |
| --- | --- | --- | --- | --- | --- |
|  | **0-1** | **2** | **3** | **4-5** | ***P-*_trend_** |
| **Microvascular complications** | | | | | |
| No macrovascular disease (N=15,104) | | | | | |
| Cases | 378 | 507 | 312 | 99 |  |
| Model 1 | 1.00 | 0.71 (0.67, 0.76) | 0.65 (0.61, 0.69) | 0.54 (0.48, 0.59) | <0.001 |
| Model 2 | 1.00 | 0.71 (0.62, 0.81) | 0.65 (0.56, 0.76) | 0.54 (0.43, 0.68) | <0.001 |
| With macrovascular disease (N=3397) | | | | | |
| Cases | 162 | 222 | 129 | 33 |  |
| Model 1 | 1.00 | 0.82 (0.75, 0.89) | 0.73 (0.65, 0.80) | 0.54 (0.46, 0.64) | <0.001 |
| Model 2 | 1.00 | 0.83 (0.67, 1.02) | 0.75 (0.59, 0.95) | 0.60 (0.41, 0.88) | 0.003 |
| *P*-interaction | 0.89 |  |  |  |  |
| **Diabetic retinopathy** | | | | | |
| No macrovascular disease (N=15,104) | | | | | |
| Cases | 149 | 224 | 139 | 46 |  |
| Model 1 | 1.00 | 0.81 (0.74, 0.89) | 0.75 (0.67, 0.83) | 0.64 (0.56, 0.75) | <0.001 |
| Model 2 | 1.00 | 0.82 (0.66, 1.01) | 0.76 (0.60, 0.96) | 0.65 (0.46, 0.91) | 0.005 |
| With macrovascular disease (N=3397) | | | | | |
| Cases | 46 | 71 | 38 | 17 |  |
| Model 1 | 1.00 | 0.94 (0.80, 1.12) | 0.78 (0.65, 0.95) | 1.04 (0.81,1.33) | 0.95 |
| Model 2 | 1.00 | 1.01 (0.69, 1.47) | 0.86 (0.55, 1.34) | 1.19 (0.67, 2.13) | 0.98 |
| *P*-interaction | 0.45 |  |  |  |  |
| **Diabetic kidney disease** | | | | | |
| No macrovascular disease (N=15,104) | | | | | |
| Cases | 188 | 245 | 153 | 39 |  |
| Model 1 | 1.00 | 0.69 (0.64, 0.76) | 0.64 (0.58, 0.71) | 0.42 (0.36, 0.49) | <0.001 |
| Model 2 | 1.00 | 0.69 (0.57, 0.84) | 0.63 (0.51, 0.79) | 0.43 (0.30, 0.61) | <0.001 |
| With macrovascular disease (N=3397) | | | | | |
| Cases | 98 | 142 | 84 | 16 |  |
| Model 1 | 1.00 | 0.87 (0.77, 0.97) | 0.79 (0.69, 0.90) | 0.44 (0.34, 0.55) | <0.001 |
| Model 2 | 1.00 | 0.86 (0.66, 1.11) | 0.79 (0.58, 1.06) | 0.47 (0.27, 0.80) | 0.01 |
| *P*-interaction | 0.84 |  |  |  |  |
| **Diabetic neuropathy** | | | | | |
| No macrovascular disease (N=15,104) | | | | | |
| Cases | 110 | 122 | 61 | 22 |  |
| Model 1 | 1.00 | 0.60 (0.53, 0.67) | 0.44 (0.38, 0.51) | 0.42 (0.34, 0.51) | <0.001 |
| Model 2 | 1.00 | 0.61 (0.47, 0.79) | 0.47 (0.34, 0.64) | 0.46 (0.29, 0.74) | <0.001 |
| With macrovascular disease (N=3397) | | | | | |
| Cases | 46 | 50 | 22 | 7 |  |
| Model 1 | 1.00 | 0.65 (0.54, 0.77) | 0.45 (0.36, 0.56) | 0.41 (0.29, 0.58) | <0.001 |
| Model 2 | 1.00 | 0.72 (0.48, 1.09) | 0.56 (0.33, 0.95) | 0.54 (0.24, 1.22) | 0.02 |
| *P*-interaction | 0.99 |  |  |  |  |

**Model 1:** unadjusted model.

**Model 2:** age (continuous, years), sex (male, female), ethnicity (White, others), education attainment (college or university degree, A/AS levels or equivalent or O levels/GCSEs or equivalent or other professional qualifications, or none of the above), Townsend Deprivation Index (continuous), sleep duration (<6, 6,8, or ≥9 hours/day), family history of CVD (yes, no), family history of hypertension (yes, no), prevalence of hypertension (yes, no), diabetes duration (continuous, years), use of diabetes medication (none, only oral medication pills, or insulin or others), HbA_1c_ (continuous, mmol/mol), use of antihypertensive medication (yes, no), use of lipid-lowing medication (yes, no), and use of aspirin (yes, no). *P*-interaction were based on Model 2.
